# Early Flowering 3 (ELF3) Inhibits Hypocotyl Phototropism in Light‐Grown *Arabidopsis* Seedlings

**DOI:** 10.1002/pld3.70107

**Published:** 2025-09-23

**Authors:** Geoffrey M. C. Cobb, Johanna Krahmer, Ganesh M. Nawkar, Alessandra Boccaccini, Sandi Paulišić, Christian Fankhauser

**Affiliations:** ^1^ Centre for Integrative Genomics, Faculty of Biology and Medicine, Genopode Building University of Lausanne Lausanne Switzerland; ^2^ Department of Plant and Environmental Sciences University of Copenhagen Frederiksberg C Denmark; ^3^ Dr. D.Y Patil Biotechnology and Bioinformatics Institute (DYPBBI) Dr. D.Y. Patil University (Deemed to be University) Pune Maharashtra India; ^4^ Department of Science and Technology for Sustainable Development and One Health Università Campus Bio‐Medico di Roma Rome Italy

**Keywords:** *Arabidopsis thaliana*, *Brachypodium distachyon*, circadian rhythms, ELF3, phototropism, PIF

## Abstract

Phototropic bending of plants towards a light source allows them to position their photosynthetic tissues to optimize light capture. In light‐grown (de‐etiolated) *Arabidopsis* seedlings, phototropic bending of the hypocotyl is inhibited by light with a high red:far‐red ratio (HRFR) and high levels of blue light (HBL). This occurs via activation of the phytochrome B (phyB) and cryptochrome 1 (cry1) photoreceptor signaling pathways. Both phyB and cry1 act upstream of PHYTOCHROME INTERACTING FACTOR (PIF) transcription factors, which are required for hypocotyl bending in light‐grown seedlings. Presently, it is not known whether other pathways are involved in the inhibition of PIF‐mediated phototropism in light‐grown seedlings. To address this, we conducted a screen to identify mutants with increased phototropic bending relative to wild type in HRFR + HBL conditions. Through this screen, we identified EARLY FLOWERING 3 (ELF3), a member of the Evening Complex (EC), as a key inhibitor of phototropic bending in green seedlings. We show that both ELF3 and LUX, another component of the EC, inhibit phototropic bending upstream of PIF4/PIF5. Furthermore, we show that phototropic bending in *Arabidopsis* seedlings is subject to circadian regulation in an ELF3‐dependent manner. Finally, we provide evidence that ELF3 in the grass *Brachypodium distachyon* also affects phototropism but in an opposite way than in *Arabidopsis*.

## 1 Introduction

Plants in the wild must often compete with neighboring plants for resources such as water, nutrients, and light. In canopy shade conditions, where a plant is completely overtopped by its neighbors, the ratio of red to far‐red light is lower (LRFR conditions), and the level of blue light is also lower (LB conditions) than in full sunlight (Fiorucci and Fankhauser 2017). In these environments, plants may bend towards available sources of light to maximize their exposure. In light‐grown (de‐etiolated) *Arabidopsis* seedlings, this phototropic bending requires the PHYTOCHROME INTERACTING FACTORS (PIFs) 4, 5, and 7 (Goyal et al. 2016; Boccaccini et al. 2020). PIF4/5/7 are transcription factors that promote the expression of *YUCCA* auxin biosynthesis genes, which are themselves required for bending (Goyal et al. 2016).

In sunny conditions, hypocotyl phototropism is weak in de‐etiolated *Arabidopsis* seedlings, presumably due to sufficient access to light (Iino 2001; Boccaccini et al. 2020). In this case, the high red:far‐red ratio (HRFR) and higher levels of blue light (HB) activate phyB and cry1 photoreceptor signaling pathways, which result in the repression of the PIFs, thereby inhibiting phototropism (Goyal et al. 2016; Boccaccini et al. 2020). PIF4/5/7 thus form a critical link between sensing of a plant's light environment and growth responses such as phototropism. Whether PIFs are regulated in additional ways to control their function in hypocotyl phototropism remains unknown.

The Evening Complex (EC) is a protein complex that plays a critical role in the function of the circadian clock in plants by repressing the expression of key genes around the end of the light photoperiod (Huang and Nusinow 2016). The EC in *Arabidopsis* has three components: EARLY FLOWERING 3 (ELF3), EARLY FLOWERING 4 (ELF4), and LUX ARRYTHMO (LUX) (Huang and Nusinow 2016). While the EC has not previously been demonstrated to affect phototropic bending, it is known to repress the transcription of PIFs at the end of long days, resulting in a daily cycle of PIF transcript levels (Nusinow et al. 2011; Murcia et al. 2022). Protein levels of PIF4/5/7 follow their own transcript levels closely (Galvao et al. 2019; Murcia et al. 2022), indicating that the EC likely plays a key role in maintaining a daily cycling of PIF protein levels. Additionally, ELF3 is known to repress the activities of PIF4 and PIF7 independently of the EC via direct protein–protein interaction, which prevents PIFs from binding to their target genes (Nieto et al. 2015; Jiang et al. 2019). As important regulators of PIFs, the EC members are good candidates for possible regulators of phototropism in light‐grown seedlings.

Historically, mutant screens in dark‐grown (etiolated) *Arabidopsis* seedlings have successfully identified many components necessary for phototropic bending. For example, the blue light photoreceptor phot1 was identified in several screens for mutants with reduced capacity for hypocotyl bending in response to unilateral blue light (Khurana and Poff 1989; Liscum and Briggs 1995). NPH3 and RPT2, important regulators of phototropic bending and direct interactors of PHOT1, were also identified in such screens (Okada and Shimura 1992; Liscum and Briggs 1995), as were the cryptochromes CRY1 and CRY2 (Ahmad et al. 1998). Phot1, NPH3, and cry1 have since been demonstrated to be important regulators of phototropism in light‐grown seedlings as well as dark‐grown seedlings (Goyal et al. 2016; Boccaccini et al. 2020). However, phototropic bending in dark‐grown seedlings does not require PIF4/5/7, meaning that these screens are insufficient to identify regulators of PIF‐mediated phototropism in light‐grown seedlings (Goyal et al. 2016).

To address this, we initiated a mutant screen to identify negative regulators of phototropism in light‐grown *Arabidopsis* seedlings. Through this screen, we identified mutants defective in phytochrome and cryptochrome signaling, confirming the importance of these pathways to the control of phototropism. We also identified a nonsense allele in *ELF3* that conferred increased phototropic bending over wild type in our screen conditions. We demonstrate that ELF3 and LUX act upstream of PIF4/5 to inhibit bending. Furthermore, we show that bending is subject to diurnal regulation, whose rhythm rapidly attenuates upon transfer to constant light conditions. Finally, given that ELF3 and the PIFs are very highly conserved among land plants, we performed an initial characterization of the role of the *ELF3* gene in regulating phototropism in the grass *Brachypodium distachyon* .

## 2 Results

### 2.1 A Mutant Screen for Negative Regulators of Phototropism in Light‐Grown *Arabidopsis* Seedlings

To identify negative regulators of phototropic bending in light‐grown seedlings, we first determined growth conditions under which wild‐type (Col‐0) seedlings do not bend towards a lateral light source, while a loss‐of‐function mutant in a known inhibitor of phototropic bending (*phyB‐9*) is able to bend. The precise conditions used for the screen are detailed in Section 4.

Using these growth conditions, we screened an M2 population of EMS‐mutagenized seeds for mutants with increased phototropic bending relative to wild type. In total, we tested approximately 27,000 individual M2 seedlings, sourced from 126 seed pools, with each seed pool containing seeds from 35 M1 plants. A small number of seedlings with increased phototropic bending were grown for at least one additional generation and retested, then backcrossed to wild type. An overview of the testing regime is shown in Figure S1B. Bending phenotypes were then assessed in the F1 and F2 backcross to determine their segregation pattern.

Using this method, we identified candidate lines with reproducible bending phenotypes, several of which are summarized in Table 1. Because phyB and cry1 are already known to negatively regulate bending (Goyal et al. 2016; Boccaccini et al. 2020), we performed a series of tests to determine whether any of our mutant lines were impacted in the phytochrome or cryptochrome signaling pathways. *phyA*, *phyB*, and *cry1* loss‐of‐function mutants have long hypocotyls in monochromatic far‐red light, monochromatic red light, and monochromatic blue light, respectively (Neff and Chory 1998). With this in mind, we assessed the hypocotyl lengths of our candidates in monochromatic red, far‐red, and blue light. All the candidate lines tested were determined to have long hypocotyls in at least one of these light conditions (summarized in Table 1).

**TABLE 1:** Summary of candidates identified in the mutant screen.

Line ID number	Hypocotyl length phenotype in monochromatic light (M3 seedlings)	Western blot result (M4 seedlings)—see Figure S6.	Sequencing result	Possible explanation for bending phenotype
I040123	Long in R + FR	PHYA expressed in light and dark		Possible phytochrome chromophore mutant
C091602	Long in R + FR	PHYA expressed in light and dark		Possible phytochrome chromophore mutant
B121320	Long in B	CRY1 is not expressed		CRY1 is not expressed
C120501	Long in B	CRY1 is not expressed		CRY1 is not expressed
C061423	Long in B	Expresses CRY1	Substitution: R525K in *CRY1*.	Probable loss‐of‐function *cry1* mutant (Ahmad et al. 1995)
C041719	Long in R	Expresses PHYB	Substitution: G1039R in *PHYB*.	Probable loss‐of‐function *phyB* mutant (Krall and Reed 2000)
B111018	Long in R	Expresses CRY1, PHYA, and PHYB	Premature stop codon at position 565 in *ELF3*.	Probable loss‐of‐function *elf3* mutant (Hicks et al. 2001)

If a candidate was found to have a hypocotyl phenotype, which mimicked a known photoreceptor mutant, we conducted western blots to determine whether that photoreceptor was expressed. In this way, we determined that two of our candidates (B121320 and C120105), which have long hypocotyls in blue light, do not express CRY1 protein (Table 1, Figure S6).

One of our candidates (C061423) had long hypocotyls in blue light but did express CRY1 protein (Table 1, Figure S6). Sequencing of the CRY1 locus in this line revealed a substitution R525K in the C‐terminal region of the protein. Interestingly, substitutions in this region are already known to result in a loss of CRY1 function (Ahmad et al. 1995), making this a likely explanation for the increased bending phenotype of this candidate. In a similar way, we identified a candidate (C041719) with long hypocotyls in red light, which expressed PHYB protein (Table 1, Figure S6). Sequencing of the phyB locus in this line revealed a substitution G1039R in the C‐terminal histidine kinase domain. Mutations in this region are known to result in a loss of phyB function (Krall and Reed 2000).

Some candidates (I040123 and C091602) had long hypocotyls in both red and far‐red light, indicating possible disruption of both phyB and phyA functions simultaneously. Insensitivity to both red and far‐red light is a characteristic of mutants, which are impaired in phytochrome chromophore biosynthesis (Goto et al. 1993). In such mutants, phyA is stable when dark‐grown seedlings are exposed to light (Parks et al. 1989; Parks and Quail 1991). Therefore, we conducted a western blot to compare phyA levels in dark‐grown and white‐light‐exposed seedlings in our candidate lines. We found that these candidates possess photostable phyA (Table 1, Figure S6).

### 2.2 A Nonsense Allele in *ELF3* Confers Increased Phototropic Bending

**FIGURE 1:**
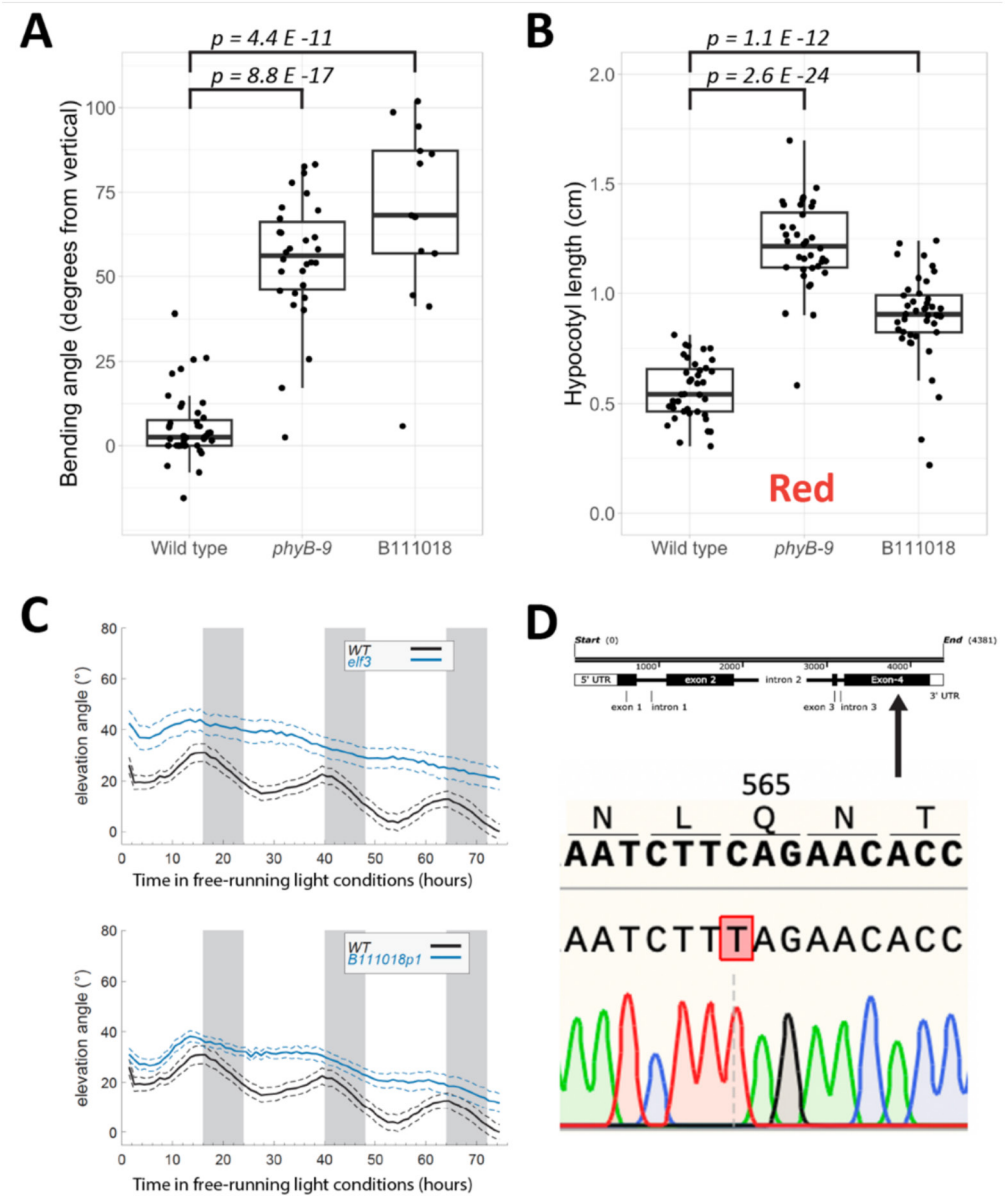
Initial characterization of the B111018 phototropic bending mutant. (A) Quantification of phototropic bending in B111018 seedlings grown in our screen conditions. Points represent the bending angles of individual seedlings; boxplots show the median bending angle and quartiles. For this experiment, seeds of candidate B111018 were from the M3 generation. Bending in the M3 generation was tested twice with similar results, and bending in the M4 generation was also tested (see Figure S3). (B) Hypocotyl lengths of 4‐day old seedlings grown in constant monochromatic red light at 14 μmol m^2^ s^−1^. Seeds from B111018 were from the M3 generation. *p*‐values in (A) and (B) are derived from Welch's *t*‐test. (C) Leaf elevation angle (angle from leaf base to tip, relative to horizontal) in free‐running light conditions of the first two true leaves on long‐day entrained WT (wild‐type), *elf3–1*, and B111018 rosettes. B111018 plants were from the M4 generation. Plants were entrained in long days (16L/8D) and were 2‐weeks old upon transfer to constant light for observation. In the graphs, the solid lines represent mean leaf elevations over time, and the dotted lines indicate the 95% confidence interval around the mean estimates. Plants were measured about once per hour. In the top part of panel (C), data from 13 wild‐type and 13 *elf3* plants are shown. In the bottom part of the panel, data from the same wild‐type individuals are shown alongside data from 12 individuals of B111018. (D) Schematic showing the location of the nonsense mutation in the *ELF3* locus in candidate B111018. This panel was created using Snapgene.

One of the candidates that we identified in our screen, B111018, exhibited increased phototropic bending over wild type in our screen conditions (Figure 1A), long hypocotyls in monochromatic red light (Figure 1B), and slightly longer hypocotyls in monochromatic far‐red and blue light (Figure S2). Candidate B111018 still expressed PHYB protein and possessed photolabile PHYA (Table 1, Figure S6). To further characterize candidate B111018, we tested it for altered leaf elevation (hyponasty), which is a process that depends on PIF4/5/7 and which follows a circadian pattern (Michaud et al. 2017). Interestingly, B111018 exhibited almost completely arrhythmic leaf hyponasty in free‐running light conditions, mimicking an *elf3‐1* control (Figure 1C) (Hicks et al. 1996). Therefore, we sequenced the *ELF3* locus in B111018. This revealed the presence of a premature stop codon at position 565 (Figure 1D). Encouragingly, there are several characterized nonsense mutations in the ELF3 gene, which all result in loss‐of‐function phenotypes (Hicks et al. 2001). Our mutant introduces a stop codon relatively close to the C‐terminus that eliminates a putative NLS in ELF3 (Liu et al. 2001).

Next, we backcrossed candidate B111018 to wild type and observed phototropic bending in F1 and F2 seedlings. Bending in the F1 seedlings was comparable to wild type, indicating that the mutation responsible for the increased bending is recessive (Figure S3A). Roughly one quarter (17/69) of the F2 seedlings bent more than the strongest‐bending seedling in the wild‐type control, indicating Mendelian segregation of the increased bending trait (Figure S3B).

A dCAPS genotyping strategy (Neff et al. 1998) was developed to differentiate between wild‐type plants and plants carrying the B111018 *elf3* mutation. This genotyping strategy was used to confirm that four F1 plants from our backcross were heterozygous for the B111018 *elf3* mutation (Figure S3C). We also genotyped four F2 seedlings, which exhibited the increased bending phenotype and compared them to four F2 seedlings, which lacked this phenotype. The selected F2 seedlings with increased bending were found to be homozygous for the B111018 *elf3* mutation, while those without the phenotype were found to be either homozygous for the wild‐type allele or heterozygous (Figure S3D). The backcrossed new *elf3* allele had a phototropic phenotype that was very similar to that of the presumptive null *elf3‐2* allele (Figure S3B) (Hicks et al. 2001; Liu et al. 2001). To determine whether a truncated ELF3 protein accumulates in B111018, we extracted proteins in 12:12‐grown plants at the end of the day, separated them on SDS‐PAGE, and conducted a western blot. This showed that a truncated ELF3 accumulates in this new allele (Figure S4). Collectively, our data indicate that this allele behaves as an *elf3* null allele having very similar leaf movement and phototropic defects as *elf3‐1* and *elf3‐2* (Hicks et al. 2001; Liu et al. 2001).

### 2.3 ELF3 and LUX Act Upstream of PIF4/5 to Control Phototropic Bending

**FIGURE 2:**
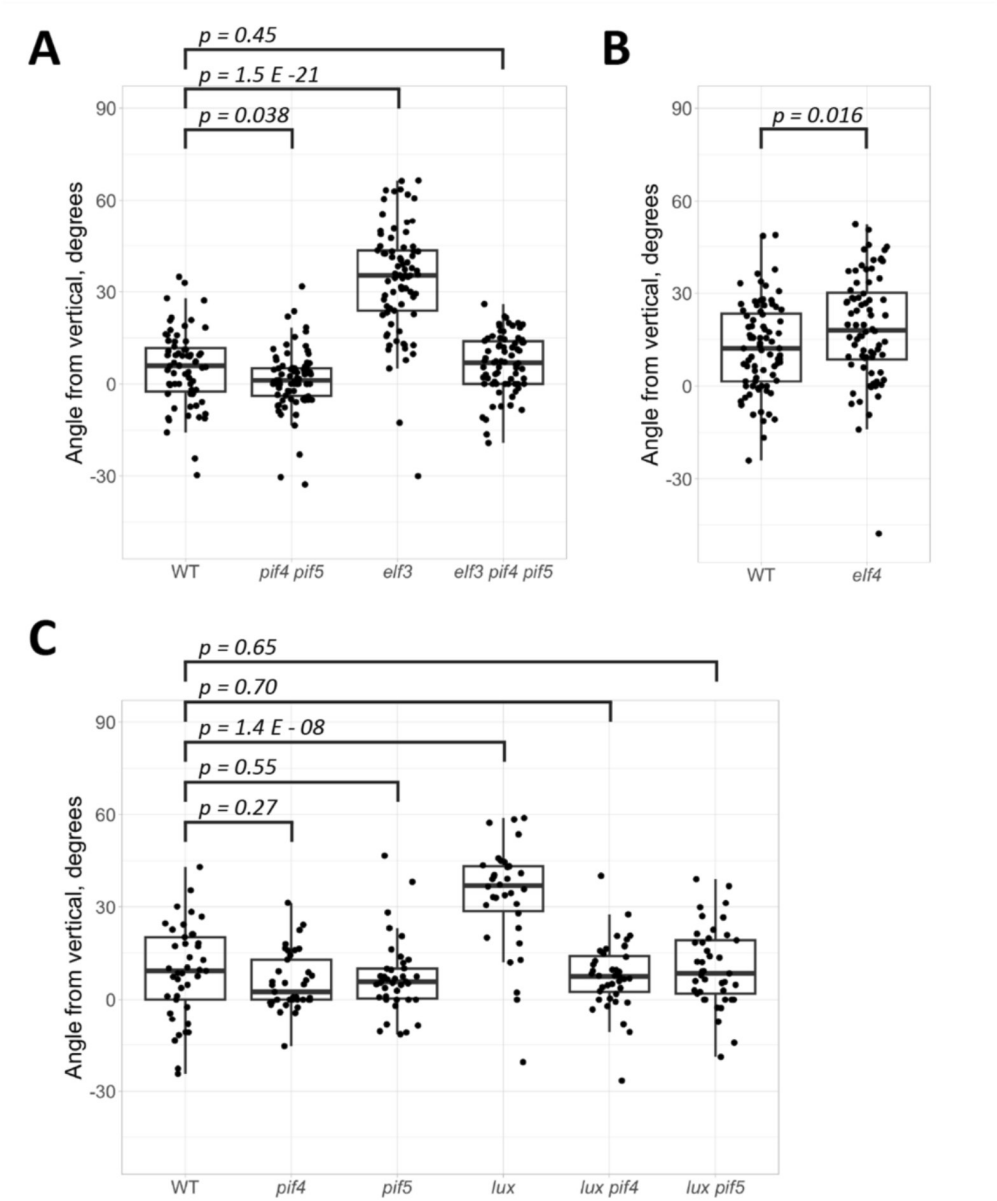
Bending assay results with *Arabidopsis* evening complex mutants. Points represent the bending angles of individual seedlings; boxplots show the median bending angle and quartiles. The *p*‐values shown are derived from Welch's *t*‐test. (A) Bending in the *elf3*, *pif4 pif5*, and *elf3 pif4 pif5* mutants. All lines in this panel are in the pTOC1::LUC background. (B) Bending in the *elf4* mutant. (C) Bending in various *lux* and *pif4/pif5* mutant combinations. All lines in this panel are in the CAB2::LUC background (Nusinow et al. 2011). Bending of each line in this figure was tested at least twice with comparable results, with the exception of *pif4* and *pif5* in the CAB2::LUC background, which were tested once.

ELF3 is a component of the EC, a transcriptional regulator that also includes ELF4 and LUX (Huang and Nusinow 2016). Therefore, we assessed phototropic bending in the *elf3‐2*, *elf4‐101*, and *lux‐4* mutants in our screen conditions. Consistent with the outcome of our screen, the *elf3‐2* allele exhibited increased phototropic bending over wild type (Figure 2). Furthermore, the phototropic bending in the *elf3 pif4 pif5* triple mutant resembled that of wild‐type seedlings, suggesting that ELF3 acts upstream of PIF4/PIF5 to control bending. The *lux* mutant showed similar behavior, with increased phototropic bending relative to wild type that was suppressed in both the *pif4* and *pif5* single mutant backgrounds (Figure 2). Surprisingly, while the bending in the *elf4* mutant appears to be slightly higher than wild type (Figure 2), this result was not always reproducible.

### 2.4 Phototropic Bending Potential in *Arabidopsis* Seedlings Follows a Circadian Rhythm

The EC is essential to the function of the circadian clock in *Arabidopsis* (Huang and Nusinow 2016). The realization that components of the EC regulate phototropism indicates that bending may be subject to circadian control. Indeed, a previous study demonstrated that long day‐entrained potato plantlets transferred to constant light maintain some rhythmicity in their ability to bend at different times of day, though this rhythm attenuates as the duration of exposure to constant light increases (Vinterhalter et al. 2015).

**FIGURE 3:**
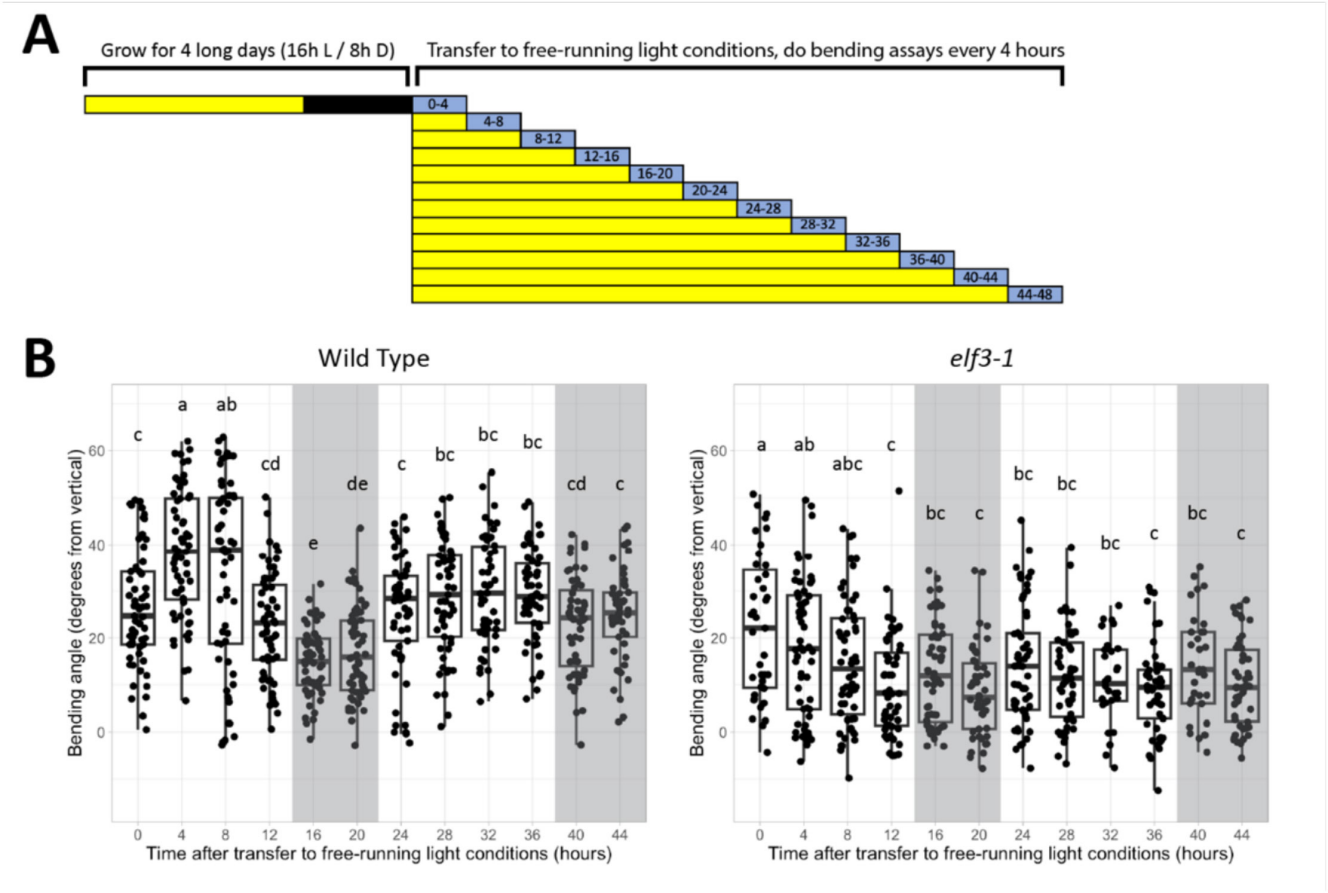
Phototropic bending at different times of day in long‐day‐entrained seedlings. (A) Schematic showing the light treatments over the course of the experiment. Seedlings were grown for 4 days in long days (16‐h light/8‐h dark) at 21°C in combined LRFR + LB light. Bending assays were then conducted every 4 h over a 48‐h timecourse, starting at dawn on the 5th day. In this schematic, yellow represents combined LRFR + LB light, black represents night, and blue represents combined LRFR + LB light supplemented with lateral blue light. (B) Hypocotyl bending of long day‐entrained seedlings transferred to constant light conditions and given lateral blue light following different durations of constant light exposure. Points represent the bending angles of individual seedlings; boxplots show the median bending angle and quartiles. Letter groupings are assigned from the results of Tukey's HSD test with a significance level of 0.05. This experiment was also conducted with wild‐type and *elf3‐2* seedlings both in the pTOC1::LUC background with a comparable result.

With this in mind, we wanted to test whether phototropic bending was also subject to circadian control in *Arabidopsis* seedlings. To observe strong bending in light‐grown wild‐type seedlings, it is necessary to grow them in combined low red:far‐red (LRFR) and low blue (LB) light. Therefore, we grew seedlings in this condition under long days, at 21°C, for 4 days. At dawn on the 5th day, seedlings were transferred to constant LRFR + LB light. At regular 4‐h intervals over a time course of 48 h, subsets of seedlings were moved to a new incubator and exposed to a lateral blue light stimulus using a blue LED array. The seedlings were then photographed after 4 h of lateral blue light exposure. A schematic of the light treatments given to each batch of seedlings is shown in Figure 3A.

We observed a peak in the bending potential of wild‐type seedlings around the middle of the first subjective day (Figure 3B). Bending decreased upon the onset of the first subjective night, but subsequently increased in anticipation of the second subjective day (Figure 3B). It then stabilized through the evening and into the second subjective night. By contrast, no such pattern was observed in *elf3* mutant seedlings (Figure 3B). Instead, bending in *elf3* seedlings decreased relative to the first few timepoints, then stabilized. These data confirm the involvement of the circadian clock in the emergence of rhythmic bending potentials.

### 2.5 Phototropic Bending in an *elf3* Mutant of *B. distachyon*

Orthologs of the *PIFs* and of *ELF3* are highly conserved among land plants (Huang et al. 2017; Possart et al. 2017; Jiang et al. 2022). Recently, efforts have been made to characterize the functions of both the *PIF* and *ELF3* genes in the model plant *B. distachyon* , a monocot (Bouche et al. 2021; Hoang et al. 2021; Jiang et al. 2022; Gao et al. 2023). With this in mind, we wanted to determine whether ELF3 inhibits phototropism in light‐grown *Brachypodium* seedlings, as it does in *Arabidopsis*. To test this, we grew wild‐type and *elf3* mutant *Brachypodium* seedlings on 1/2 MS medium in the dark for 4 days, then moved them to long days in white light. On the second day in the light, the plants were put into black boxes which only allowed light to enter from one side. Bending of the seedlings towards the lateral light (defined as the angle of the coleoptile or tip of the first leaf relative to vertical) was monitored over several successive days.

**FIGURE 4:**
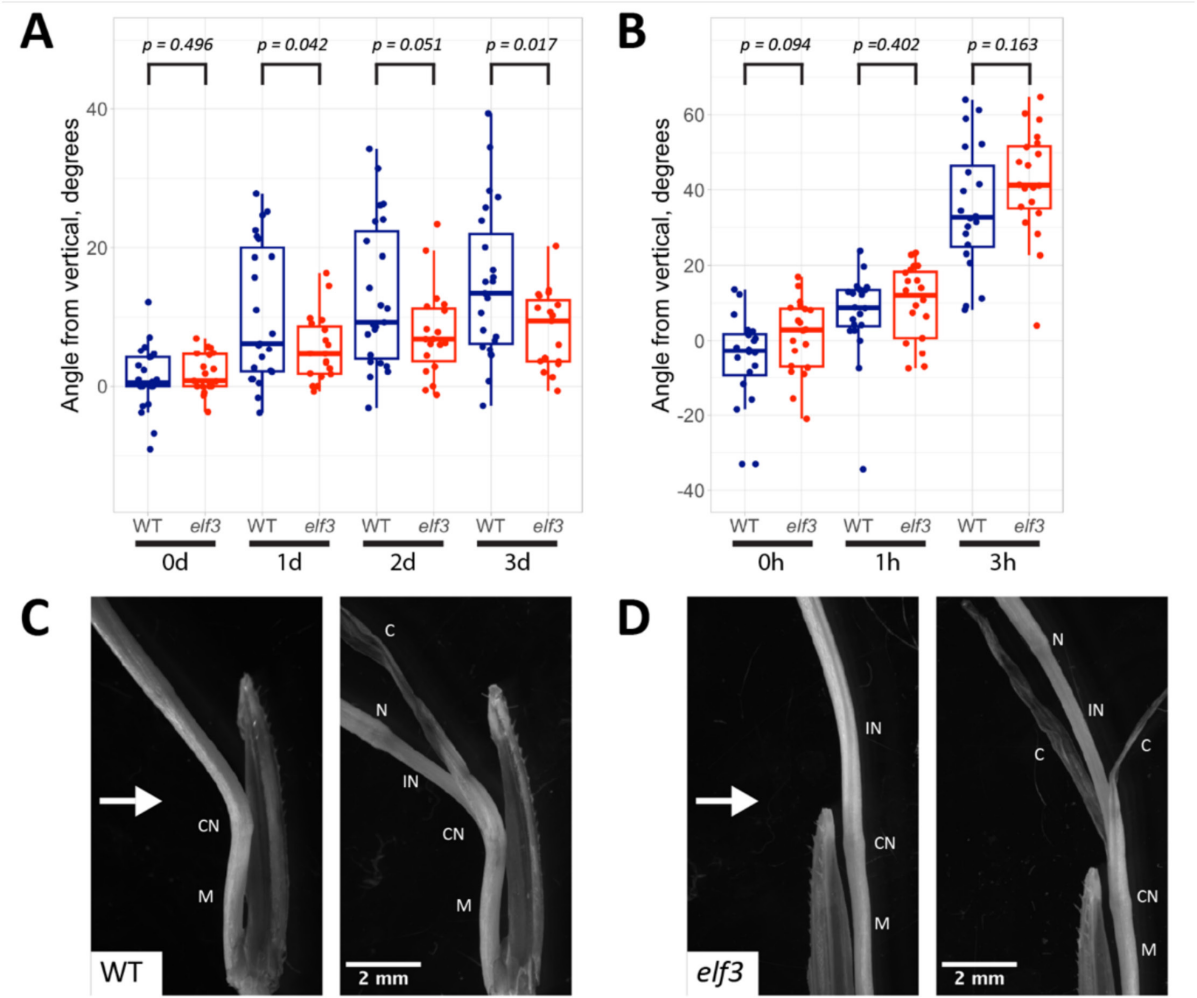
Phototropic bending in *Brachypodium* plantlets. (A) Phototropic bending in light‐grown *Brachypodium* seedlings over the course of 3 days. Points represent the bending angles of individual plantlets; boxplots show the median bending angle and quartiles. *p*‐values are derived from Welch's *t‐*test. This experiment was conducted twice with comparable results. (B) Phototropic bending in dark‐grown *Brachypodium* seedlings exposed to 2‐μmol m^−2^ s^−1^ lateral blue light. *p*‐values are derived from Welch's *t*‐test. (C) Photograph of a wild‐type *Brachypodium* seedling, showing the tissues involved in phototropic bending. The direction of the lateral light source is denoted by the white arrow. The left panel shows an intact seedling, while the right panel shows the same seedling with the coleoptile peeled back to reveal underlying structures. The labels denote the following structures: mesocotyl (“M”), coleoptile node (“CN”), first internode (“IN”), second node (“N”), and the coleoptile (“C”). (D) Photograph of an *elf3* mutant *Brachypodium* seedling, intact (left) or with the coleoptile peeled back (right).

Interestingly, we observed a tendency of the *elf3* mutant to bend less than the wild type, which is the opposite of the bending phenotype for the *elf3* mutant in light‐grown *Arabidopsis* seedlings (Figure 4A). Though bending was weak overall and quite variable for both genotypes, this difference was reproducible. To determine whether the *Brachypodium elf3* mutant has a general bending defect, which could account for this, we also compared the bending phenotypes of dark‐grown wild‐type and *elf3* seedlings exposed to 2‐μmol m^−2^ s^−1^ lateral blue light. In these conditions, bending between the wild type and *elf3* mutant was comparable, suggesting that the reduced bending phenotype of *elf3* is specific to light‐grown seedlings (Figure 4B).

Because the anatomy of dicots (e.g., *Arabidopsis*) and monocots (e.g., *Brachypodium*) is markedly different, we conducted light microscopy to determine which tissues were responsible for the phototropic bending in light‐grown *Brachypodium* plantlets. When seedlings are young, it is difficult to distinguish between the bending of the coleoptile and other seedling structures, because they are enveloped by the coleoptile. Therefore, we observed plantlets that had been exposed to lateral light for an extended period (8 days). At this stage, the plantlets generally have a well‐developed stem and two visible leaves. In these specimens, much of the bending seems to occur at either the coleoptile node or the first node above it (see example in Figure 4C). Curvature of the first internode region may also contribute to the bending, though this is not always present (see example in Figure 4D). This suggests that phototropism in light‐grown *Brachypodium* is a complex behavior that can involve growth responses across multiple organs.

## 3 Discussion

Positive phototropism in plants is thought to be important for allowing them to optimize their light exposure in light‐limited environments (Iino 2001; Fiorucci and Fankhauser 2017). In light‐grown *Arabidopsis* seedlings, phototropism of the hypocotyl is inhibited by the phytochrome and cryptochrome signaling pathways, which repress the action of PIFs 4/5/7, thus preventing bending in conditions when it is not needed (Goyal et al. 2016; Boccaccini et al. 2020). In this study, we sought to identify additional negative regulators of phototropism in light‐grown seedlings using a forward genetics approach. We identified several candidate lines with increased phototropic bending relative to wild type in our screening conditions. Most of these lines were subsequently found to be defective in various aspects of phytochrome or cryptochrome signaling (Table 1), thus highlighting the critical importance of these signaling pathways for the regulation of phototropism. One of our candidates possesses a nonsense mutation at the *ELF3* locus. The phenotype of this new allele is like that of *elf3* loss of function mutants (Figures 1C and S3B). A truncated ELF3 protein accumulates in this mutant, but it lacks the putative NLS of ELF3 (Figure S4) (Liu et al. 2001), confirming the importance of the C‐terminus of ELF3 that was previously shown to be required for normal nuclear import and ELF3 function (Herrero et al. 2012). Because ELF3 is also known to act antagonistically to the PIFs, the discovery of this mutant further emphasizes the central role that the PIFs play in regulating phototropism in light‐grown seedlings.

ELF3 is a member of the tripartite EC, which is known to be important for the diurnal rhythm of *PIF4/5* transcription (Nusinow et al. 2011; Murcia et al. 2022). This complex contains two other members, ELF4 and LUX. LUX is a transcription factor and is essential for allowing the EC to interact with its target promoters (Helfer et al. 2011; Silva et al. 2020). The molecular function of ELF4 is somewhat less clear. It has been proposed to enhance the DNA binding affinity of the EC (Silva et al. 2020) and is also required for normal EC function (Nusinow et al. 2011). With this in mind, we decided to test the bending phenotypes of the *lux* and *elf4* mutants. The *lux* mutant exhibited increased bending over wild type in our screen conditions, mimicking the phenotype of the *elf3* mutant (Figure 2). Our data is therefore consistent with the EC modulating hypocotyl phototropism in light‐grown seedlings. Moreover, given the known function of the EC in the regulation of PIFs, we show that the enhanced phototropic response of *elf3* and *lux* depends on the PIFs. In contrast to *elf3* and *lux*, the *elf4* mutant did not reproducibly bend more than wild type (Figure 2). The *elf4‐101* allele used in our study was previously reported to have undetectable transcript levels, indicating that it is likely to be a true null (Khanna et al. 2003). As such, residual *ELF4* expression probably does not account for this observation. Therefore, we propose two alternative explanations. First, *Arabidopsis* possesses several homologs of *ELF4* known as *ELF4‐LIKE* (*EFL*) genes, which are reported to act redundantly to control flowering time (Lin et al. 2019). Of those, EFL1 is the only one that can restore the ELF4 circadian phenotype, making it the most likely candidate (Kolmos et al. 2009). Second, we note that the *elf4* mutants are reported to have stronger phenotypes in short days than in long days (Doyle et al. 2002; Hazen et al. 2005). Our bending assays were conducted with seedlings grown in long days. Therefore, it is possible that a phenotype for *elf4* mutants might be detectable in short days. In summary, our data indicate the EC limits hypocotyl phototropism in light‐grown seedlings by modulating the PIFs.

The role of ELF3 and LUX in modulating the strength of phototropic bending suggests that phototropism is regulated in a circadian manner. We tested this in shade‐mimicking conditions when hypocotyl phototropism is robust. Seedlings grown in shaded long days and subsequently transferred to constant light conditions maintain a rhythmic bending potential, with the maximum bending response occurring in the middle of the subjective day (4–8 h after subjective dawn) and decreasing during the subjective night (Figure 3B). Notably, this pattern also correlates with published data about PIF4/5/7 protein levels, which themselves cycle in a circadian manner (Nozue et al. 2007; Galvao et al. 2019). Consistent with circadian‐regulated phototropism potential, this response was lost in the *elf3* mutant. Lack of circadian rhythmicity in *elf3* mutants is also observed for other phenotypes including leaf (Figure 1C) and cotyledon movements and hypocotyl growth (Nozue et al. 2007). Importantly, *elf3* mutants are also known to exhibit altered circadian patterns of *PIF* transcription and PIF protein levels, further suggesting that cycling of PIF protein levels might be the mechanism responsible for the circadian gating of bending potential (Nusinow et al. 2011; Murcia et al. 2022; Zhu et al. 2022).

As previously discussed, the rhythmic bending potential in wild‐type *Arabidopsis* seedlings closely resembles that of potato plantlets (Vinterhalter et al. 2015), indicating that this behavior may be found across diverse plant lineages. Interestingly, the amplitude of this rhythm decreases after the first subjective day of free‐running light conditions in both *Arabidopsis* and potato (Figure 3B) (Vinterhalter et al. 2015). This suggests that constant entrainment by day/night cycles is required to maintain the rhythmic bending potential in the long term. In our experiments, the attenuation of this pattern on the second subjective day in constant light is likely exacerbated by the fact that our seedlings were grown in LRFR conditions, which are known to decrease the amplitude of circadian rhythms in free‐running conditions (Fraser et al. 2021). However, we were still able to see evidence for circadian regulation of phototropism despite this limitation.

*ELF3* homologs are conserved between the dicots (including the model species *Arabidopsis thaliana* ) and the monocots (including the model species *B. distachyon* ), which together make up the two largest groups within the flowering plants (Huang et al. 2017). Furthermore, complementation studies suggest that the *ELF3* homolog in *Brachypodium* functions similarly to the native gene in *Arabidopsis* (Huang et al. 2017), and recent research indicates that the EC functions similarly in *Brachypodium* as it does in *Arabidopsis* in the control of flowering time (Gao et al. 2023). Therefore, we hypothesized that ELF3 might also regulate phototropism in monocots. To test this, we conducted bending assays with a previously described *Brachypodium elf3* mutant (Bouche et al. 2021). Bending in the *Brachypodium elf3* mutant was actually slightly lower than that of wild type, and overall bending was relatively weak in both lines (Figure 4A). The reasons for this difference in phototropic behavior between *Arabidopsis* and *Brachypodium elf3* mutants are not clear. We found that phototropic bending in *Brachypodium* plantlets is derived largely from the nodes (Figure 4C), which are not analogous structures to the *Arabidopsis* hypocotyl. As such, the organs and tissue types involved in the bending response differ between both species. Notably, gravitropic bending (bending in response to gravity) has already been shown to require differential growth within the nodes of several other monocot species (Zhang et al. 2011; Clore 2013), indicating that node‐based movements may be a common feature of tropic behaviors in monocots. Moreover, there is some evidence to suggest that the roles of the PIFs may differ between *Arabidopsis* and *Brachypodium*. RNAi knockdown of *BdPIL1* and *BdPIL3*, which are orthologs of *Arabidopsis PIF1* and *PIF3*, results in longer stem internode lengths and lower leaf chlorophyll content in *Brachypodium* (Hoang et al. 2021). By contrast, analysis of various *pif* mutants in *Arabidopsis* suggests that they have the opposite effect on both phenotypes. For example, the *pif1/3/4/5* quadruple mutant in *Arabidopsis* has reduced internode length in the *ath1* mutant background (Ejaz et al. 2021), and the *pif3* and *pif4/5* mutations in *Arabidopsis* cause increased chlorophyll accumulation in leaves (Li et al. 2021). Given this, we consider the possibility that the reduced phototropic bending in the *Brachypodium elf3* mutant might be due to differences in the roles of the PIFs in *Brachypodium* as compared to *Arabidopsis*. Further work is required to determine whether this is the case.

## 4 Materials and Methods

### 4.1 Plant Materials Used

All *A. thaliana* lines used in this study are of the Columbia‐0 (Col‐0) ecotype. The EMS‐mutagenized M2 seed populations (Podolec et al. 2021) were donated by the lab of Roman Ulm (University of Geneva). The *elf3‐1* (Hicks et al. 2001), *elf3‐2* (Hicks et al. 2001), *lux‐4* (Hazen et al. 2005), *elf4‐101* (Khanna et al. 2003), *pif4‐101* (Lorrain et al. 2008), *pif5‐3* (Fujimori et al. 2004), *phyB‐9* (Reed et al. 1993), *cry1‐304* (Thum et al. 2001), and *phyA‐211* (Nagatani et al. 1993), *elf3‐2*, *pif4‐101 pif5‐3*, and *elf3‐2 pif4‐101 pif5‐3* lines in the pTOC1::LUC background, *lux‐4, lux‐4 pif4‐101*, and *lux‐4 pif5‐3* lines in the CAB2::LUC background (Nusinow et al. 2011) have been described previously. The wild type, *pif4‐101*, and *pif5‐3* lines in the CAB2::LUC background were generated by crossing. The *B. distachyon* accession used in this study was Bd21‐3; the *elf3* mutant was described previously (Bouche et al. 2021).

### 4.2 Phototropic Bending Assay for the Mutant Screen

*Arabidopsis* seeds were surface sterilized by soaking in 70% ethanol + 0.01% Triton‐X‐100 and agitating for 5 min, then rinsing them with 100% ethanol. Seeds were allowed to dry and were then sown on plates containing 1/2 Murashige and Skoog (MS) medium containing 0.8% agar. The plates were then kept in the dark at 4°C for 5 days. Next, the plates were transferred to a growth chamber. The plates were mounted in black boxes that covered the sides and the bottom of the plate so that they only received light from above (Figure S1A). The seedlings were grown in 120‐μmol m^2^ s^−1^ cool white light at 21°C in long days (16L/8D) for 4 days. On the beginning of the 5th day, the black box was rotated such that the seedlings no longer received light from above but rather from the side (Figure S1A). Phototropic bending was assessed after 6 h of lateral light exposure. In these conditions, wild‐type seedlings do not bend towards the light‐exposed side of the plate, but *phyB‐9* seedlings do bend (Figure S1B). The light conditions used to grow our seedlings are further detailed in Figure S5.

### 4.3 Sequencing of the *PHYB*, *CRY1*, and *ELF3* Loci

Fragments of the *PHYB*, *CRY1*, and *ELF3* loci were amplified from genomic DNA using the primers listed in Table S1. These same primers were then used for Sanger sequencing of the PCR fragments. Samples were sent to Microsynth AG (https://www.microsynth.com/home‐ch.html) for sequencing.

### 4.4 Measuring Hypocotyl Length in Monochromatic Light Conditions

Seeds were sterilized as described above, then plated on 1/2 MS medium and stratified in the dark for 5 days at 4°C. Seeds were then exposed to 4 h of white light to induce germination, before being returned to the dark for 20 h. Then, the plates were mounted vertically and either kept in the dark or exposed to different monochromatic light conditions: red (14.13 μmol m^−2^ s^−1^), far‐red (4.76 μmol m^−2^ s^−1^), or blue (8.04 μmol m^−2^ s^−1^), as measured with an IL1400A photometer. Hypocotyl length was measured on the 5th day after transfer to the monochromatic light conditions.

### 4.5 Leaf Movement Assay

Seeds were sterilized, then stratified on moist filter paper in the dark at 4°C for 5 days. Then, the seeds were transferred to pots of soil. This consisted of potting soil mixed in a 2:1 ratio with vermiculite. A 0.5‐cm‐thick layer of topsoil (Compo Sana Terreau pour semis et plantes aromatiques, from OBI) was added on top, and the seeds were placed on the surface of the topsoil. Next, a thin layer of powdered charcoal was spread over the remaining surface of the pot. A template was used to prevent the seeds from being covered by the charcoal. Then, plants were grown in white light (~50‐μmol m^−2^ s^−1^ PAR, measured with the IL1400A photometer), in long days (16‐h light/8‐h dark), with a day temperature of 21.5°C and a night temperature of 20°C. On the 14th day of growth, the pots were transferred to a ScanAlyzer HTS machine for leaf tracking (for more details about the device, see Dornbusch et al. 2012). Within the ScanAlyzer, plants continued to grow in the same day/night light cycles for 1 day. From dawn on the next day, seedlings were kept in constant white light. Leaf movements were tracked by the ScanAlyzer machine over the course of the following 3 days.

### 4.6 Circadian Phototropism Experiment

Seeds were sterilized and plated on 1/2 MS medium (containing 1.6% agar), which was covered in nylon mesh (Merck reference NY6H00010). The mesh was intended to reduce friction between the seedlings and the surface of the plate. The seeds were stratified at 4°C for 3 days. Then, the plates were transferred to a growth chamber. The plates were mounted vertically in black boxes such that light could only enter from the top. Seedlings were grown in combined low red:far‐red (LRFR) and low blue light (LB), using cool white growth lights supplemented with far‐red LEDs, and by covering the plates with a yellow filter. The light had a PAR (400–700 nm) intensity of ~69 μmol m^−2^ s^−1^, as measured with a PG200N spectrometer. Seedlings were grown in these conditions for four long days (16‐h light/8‐h dark). More information about the light conditions used is shown in Figure S5. On the 5th day, the side of the box was opened and seedlings were exposed to lateral light, which consisted of the ambient light in the incubator, supplemented with additional blue LEDs. This gave a total lateral blue intensity (400–500 nm) of ~25 μmol m^−2^ s^−1^. Different batches of seedlings were exposed to lateral blue light starting at different timepoints in 4‐h intervals over the course of 2 days. Phototropic bending was assessed after 4 h of exposure to lateral blue light.

### 4.7 Bending Assay for *B. dystachion* Plantlets

Prior to sterilizing seeds, the awns were removed to allow seedlings to grow more freely. Seeds were then surface‐sterilized. First, the seeds were rinsed with water. Then, they were soaked in 70% ethanol and 0.01% Triton X‐100 for 30 s, then rinsed again with water, then soaked in 1.4% sodium hypochlorite for 4 min. Finally, seeds were washed with water three times and placed onto moistened filter paper in petri dishes. The petri dishes were then wrapped in aluminum foil and left to stratify in the dark at 4°C for 6 days. After stratifying, seeds were transferred to 1/2 MS medium (containing 0.8% agar), with the embryo submerged in the medium and the top of the seed protruding. The seeds were exposed to monochromatic red light (~15 μmol m^2^ s^−1^ as measured with an IL1400A photometer) for 1 h to induce germination, and were then allowed to grow in the dark at 18°C for 4 days. To assess bending in dark‐grown seedlings, the seedlings were exposed to 2‐μmol m^2^ s^−1^ lateral blue light and monitored over the course of a day. To assess bending in light‐grown seedlings, seedlings were first transferred to white light for one long day (16‐h light/8‐h dark) in a black box that only allowed light exposure from above. The black box was then rotated so that seedlings were only exposed to lateral white light from the start of the second day. Seedlings were then monitored over the course of several days.

To assess which tissues contribute to bending in *Brachypodium*, we imaged seedlings using a Leica M205 FCA stereomicroscope.

### 4.8 Western Blots

Seeds were plated on 1/2 MS medium and stratified in the dark at 4°C. Germination was induced by exposing seedlings to white light at 21°C for 6 h, before transferring them back to the dark to grow for 4 days at 21°C. On the 5th day, seedlings were either kept in the dark or exposed to 6 h of white light prior to harvesting. For ELF3 detection, seedlings were grown in 12‐h light:12‐h dark conditions for 7 days and collected at ZT12. Seedlings were harvested into 1.5‐mL Eppendorf tubes (dark‐grown seedlings were harvested under a green light). The samples for photoreceptor detection were ground manually in 2× FSB buffer (20% glycerol, 4% SDS, 0.02% Bromophenol Blue, 0.125‐M Tris, and 10% beta‐mercaptoethanol) using small pestles, and the samples for ELF3 detection were first frozen in liquid nitrogen, ground to powder, and then resuspended in 2× FSB buffer. Samples were heated at 95°C for 10 min and centrifuged at > 16,000 rcf for 10 min, and the supernatant was transferred to a fresh set of tubes.

Proteins were separated on 4%–15% precast Mini‐PROTEAN TGX gels (BioRad). After electrophoresis, proteins were transferred to nitrocellulose membranes using the BioRad TransBlot Turbo transfer system (mixed molecular weight program, 7 min). Total protein content in each lane was assessed by staining the membranes with Ponceau S. Next, the membranes were blocked with 1× PBS buffer containing 5% milk and 0.1% Tween‐20. Blotting for PHYB was done using the BA2 primary antibody, and PHYA was done using the AA1‐3 primary antibody (Shinomura et al. 1996). Both were then detected using an HRP‐coupled anti‐mouse secondary antibody (Promega W402B). CRY1 was probed using a primary antibody (Lin et al. 1996), and ELF3 was probed using native anti‐ELF3 antibody (Agrisera AS184168), and both were detected using an HRP‐coupled anti‐rabbit secondary antibody (Promega W401B). Tubulin was used as a loading control and was probed against the N‐terminal region of human Tubulin A1A (Abiocode M0267‐1a), followed by an HRP‐coupled anti‐mouse secondary antibody. HRP‐derived chemiluminescence was detected using an ImageQuant LAS4000 CCD camera system. In order to probe the same membrane with multiple antibodies, membranes were stripped between antibody treatments with a solution containing 1.5% glycine and 0.05% Tween‐20, adjusted to a pH of 2.5.

### 4.9 Summary of Statistical Methods Used

Welch's *t*‐test was used to compare hypocotyl length and bending angle values between two populations. *p*‐values from this test were calculated using the T.TEST function in Microsoft Excel (version 16.78.3 for Mac). Tukey's HSD test was used to compare bending angles between multiple populations (see Figure 3). An alpha value of 0.05 was defined as the threshold for significance. This test was performed using the Agricolae package (version 1.3‐7) in R (version 4.4.2).

### 4.10 Gene Accession Numbers Used in This Study

**Table:** 

Gene name	Locus ID
*PHYA*	AT1G09570
*PHYB*	AT2G18790
*CRY1*	AT4G08920
*ELF3*	AT2G25930
*ELF4*	AT2G40080
*LUX*	AT3G46640
*PIF4*	AT2G43010
*PIF5*	AT3G59060
*PIF7*	AT5G61270
*Brachypodium ELF3*	Bradi2g14290

## Author Contributions

Conceptualization: C.F. Formal analysis: G.M.C.C, J.K., G.M.N., A.B., and C.F. Funding acquisition: C.F., J.K., G.M.N., and A.B. Investigation: G.M.C.C., J.K., G.M.N., A.B., and S.P. Project administration: C.F. Validation: G.M.C.C., J.K., G.M.N., A.B., S.P., and C.F. Visualization: G.M.C.C. and J.K. Supervision: C.F. Writing – original draft preparation: G.M.C.C. and C.F. Writing – review and editing: G.M.C.C., J.K., G.M.N., A.B., S.P., and C.F.

## Conflict of Interest

The authors declare no conflicts of interest.

## Peer Review

The peer review history for this article is available in the Supporting Information for this article.

## Supporting information




**Data S1:** Peer review.

**Figure S1:** Overview of the phototropic bending mutant screen. (A) A schematic showing the setup of the bending assay in light‐grown seedlings. Left: Seedlings were grown on vertically mounted plates for four long days (16‐h light/8‐h dark) with only the top of the plate exposed to light. Right: For the bending assay, the box was rotated so that the plates were exposed to light from one side only. (B) A summarized workflow of the mutant screen. (C) Example photographs of controls (wild type and *phyB‐9*) after the bending assay. *phyB* mutants bend towards the light‐exposed side of the plate (to the left in this example), while the wild type does not bend.
**Figure S2:** Hypocotyl lengths of B111018 seedlings grown in monochromatic light conditions. Points represent the lengths of individual seedlings; boxplots show the median length and quartiles. (A) Hypocotyl lengths in far‐red light (4.76 μmol m2 s^−1^). (B) Hypocotyl lengths in blue light (8.04 μmol m2 s^−1^). (C) Hypocotyl lengths in the dark. The reported *p*‐values are derived from Welch's *t*‐test.
**Figure S3: (see previous page).** Bending in B111018 backcrosses, and genotyping B111018. (A) Quantification of phototropic bending in seedlings of B111018 backcrossed to wild type (F1 generation). Points represent the bending angles of individual seedlings; boxplots show the median bending angle and quartiles. Seedlings were grown in our screen conditions for 5 days, and the bending assay began at dawn on the 6th day. B111018 seedlings (M3 generation) are included as a reference. (B) Phototropic bending in seedlings of B111018 backcrossed to WT (F2 generation). *elf3‐2* and B111018 seedlings (M4 generation) are included for reference. Note: *p*‐values in (A) and (B) are derived from Welch's *t*‐test. (C) Genotyping of B111018 (M3 and F1 backcross). PCR was performed with primers GC146 and GC147, using genomic DNA as a template. The PCR product was either undigested (“U”) or digested with XmnI (“D”). The samples were run on a 3% agarose gel at 80 V for ~30 min. The PCR product from WT plants digests to 155 + 26‐bp fragments, while the 181‐bp PCR product from the B111018 mutant is not digested. (D) Genotyping of B111018 backcross F2 seedlings. Four F2 seedlings with the increased bending phenotype and four F2 seedlings without this phenotype were selected and genotyped. Top: Sample image of a plate after the bending assay, with some of the seedlings selected for genotyping. Bending (red) and nonbending (blue) seedlings are highlighted. Bottom: Genotyping result, including the seedlings shown in the image of the plate.
**Figure S4:** ELF3 protein accumulation in the B111018 allele. Seedlings were grown in 12‐h light: 12‐h dark conditions for 7 days. Samples were collected at ZT12. Membrane was probed using native anti‐ELF3 antibody (Agrisera AS184168) and antitubulin (loading control). ELF3 is indicated with an arrow, and asterisks indicate unspecific bands. This experiment was done three times with similar results.
**Figure S5:** Example spectra of light conditions used in our experiments. (A) Light spectrum used for the standard bending assay. (B) Light spectra used for growing seedlings (top) and for inducing phototropic bending (bottom) during the circadian phototropism experiment shown in Figure 4.
**Figure S6:** Western blots for PHYA, PHYB, and CRY1 in our candidate lines. (A) Table summarizing the candidate lines, the blots in which they are represented, and our conclusions from the western blots shown here. (B) The western blots. Seedlings were grown in the dark for 5 days. They were then either kept in the dark (labelled “D”) or were exposed to 6‐h WL (labelled “L”) prior to protein extraction. A color photograph of the membranes stained with Ponceau is shown to give an indication of loading. In cases where a single membrane was probed using multiple antibodies (e.g., blots 1, 2, and 4), it was stripped between probing with the first and second antibody. For more information, see Materials and Methods. Lanes marked with a “**” indicate mutant lines, which were not included in our final candidate list. Orange areas in Blot #1 are indicators of overexposure marked by the ImageQuant LAS4000 imaging machine.
**Table S1:** Primers used in this study.

## Data Availability

The data that support the findings of this study are available from the corresponding author upon reasonable request.
