# Supplementary material for: Early Flowering 3 (ELF3) Inhibits Hypocotyl Phototropism in Light‐Grown Arabidopsis Seedlings
Source: Plant Direct. 2025 Sep 23;9(9):e70107. doi: 10.1002/pld3.70107 (PMC12455371; doi:10.1002/pld3.70107)
Supplement: Supplementary file 2 — Figure S1: Overview of the phototropic bending mutant screen. (A) A schematic showing the setup of the bending assay in light‐grown seedlings. Left: Seedlings were grown on vertically mounted plates for four long days (16‐h light/8‐h dark) with only the top of the plate exposed to light. Right: For the bending assay, the box was rotated so that the plates were exposed to light from one side only. (B) A summarized workflow of the mutant screen. (C) Example photographs of controls (wild type and phyB‐9) after the bending assay. phyB mutants bend towards the light‐exposed side of the plate (to the left in this example), while the wild type does not bend. Figure S2: Hypocotyl lengths of B111018 seedlings grown in monochromatic light conditions. Points represent the lengths of individual seedlings; boxplots show the median length and quartiles. (A) Hypocotyl lengths in far‐red light (4.76 μmol m2 s−1). (B) Hypocotyl lengths in blue light (8.04 μmol m2 s−1). (C) Hypocotyl lengths in the dark. The reported p‐values are derived from Welch's t‐test. Figure S3: (see previous page). Bending in B111018 backcrosses, and genotyping B111018. (A) Quantification of phototropic bending in seedlings of B111018 backcrossed to wild type (F1 generation). Points represent the bending angles of individual seedlings; boxplots show the median bending angle and quartiles. Seedlings were grown in our screen conditions for 5 days, and the bending assay began at dawn on the 6th day. B111018 seedlings (M3 generation) are included as a reference. (B) Phototropic bending in seedlings of B111018 backcrossed to WT (F2 generation). elf3‐2 and B111018 seedlings (M4 generation) are included for reference. Note: p‐values in (A) and (B) are derived from Welch's t‐test. (C) Genotyping of B111018 (M3 and F1 backcross). PCR was performed with primers GC146 and GC147, using genomic DNA as a template. The PCR product was either undigested (“U”) or digested with XmnI (“D”). The samples were run on a 3% agarose [file PLD3-9-e70107-s002.pdf]

ELF3 regulates positive phototropism in light-grown *Arabidopsis* seedlings.

Geoffrey M C Cobb, Johanna Krahmer, Ganesh M Nawkar, Alessandra Boccaccini, Sandi Paulišić and Christian Fankhauser.

Supplementary material

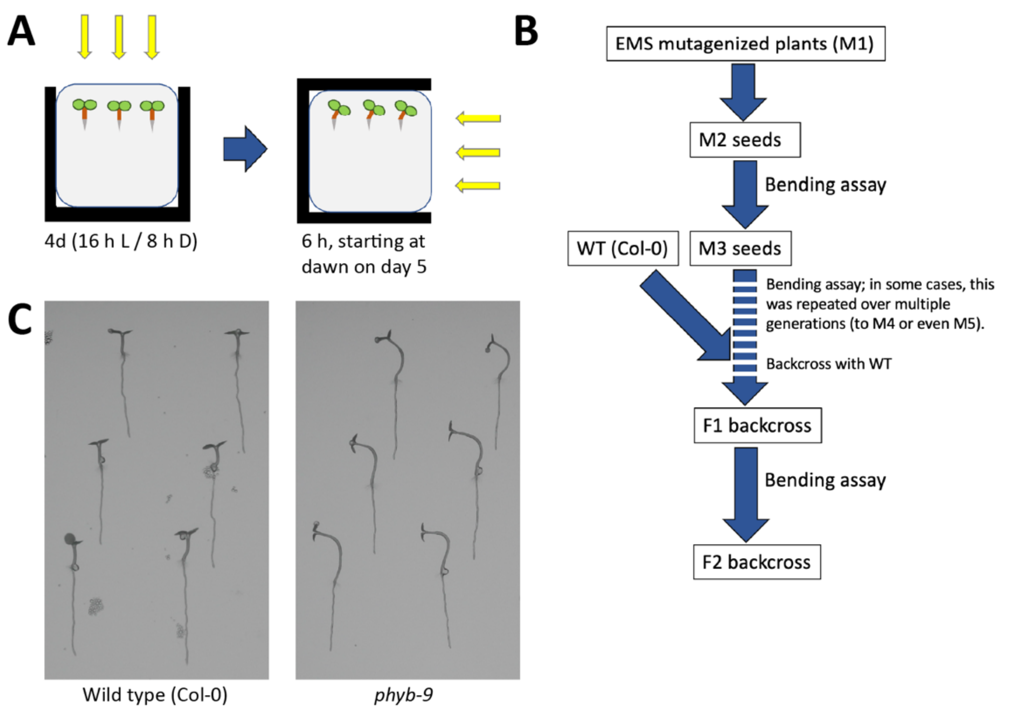

Figure S1. Overview of the phototropic bending mutant screen. (A) A schematic showing the setup of the bending assay in light-grown seedlings. Left: Seedlings were grown on vertically mounted plates for 4 long days (16 h light / 8 h dark) with only the top of the plate exposed to light. Right: For the bending assay, the box was rotated so that the plates were exposed to light from one side only. **(B)** A summarized workflow of the mutant screen. **(C)** Example photographs of controls (wild type and *phyB-9*) after the bending assay. *phyB* mutants bend towards the light-exposed side of the plate (to the left in this example), while the wild type does not bend.

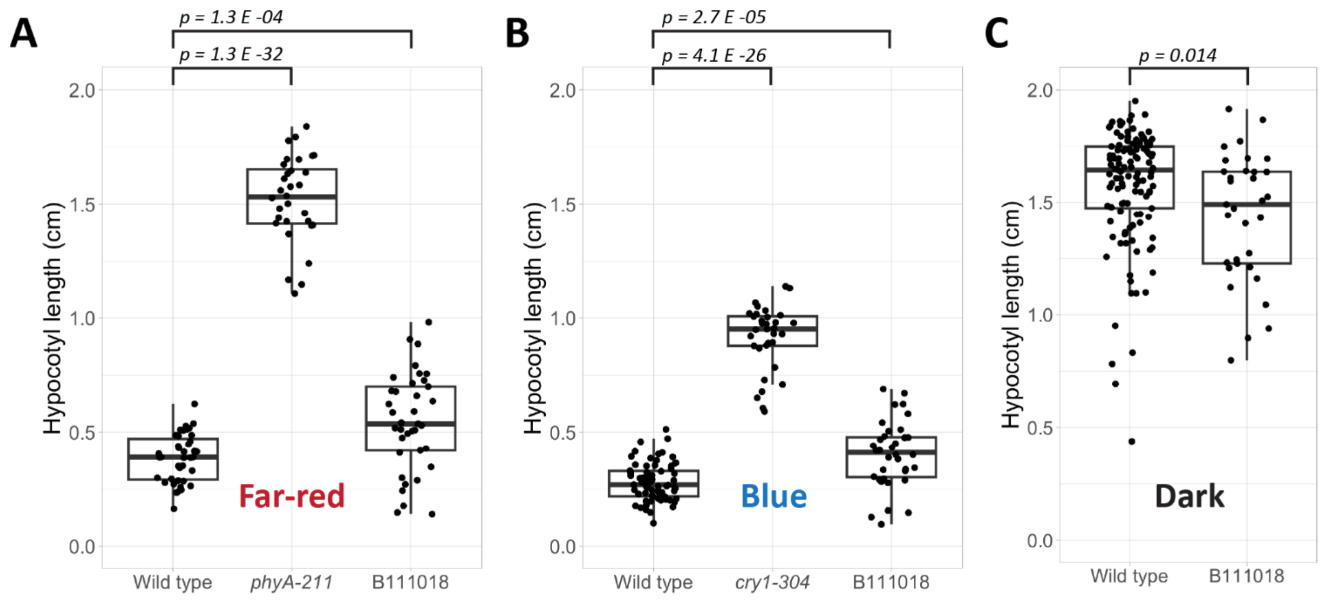

Figure S2. Hypocotyl lengths of B111018 seedlings grown in monochromatic light conditions. Points represent the lengths of individual seedlings; boxplots show the median length and quartiles. (A) Hypocotyl lengths in far-red light (4.76 µmol m^2 s^{-1}). (B) Hypocotyl lengths in blue light (8.04 µmol m^2 s^{-1}). (C) Hypocotyl lengths in the dark. The reported p-values are derived from Welch's t-test.

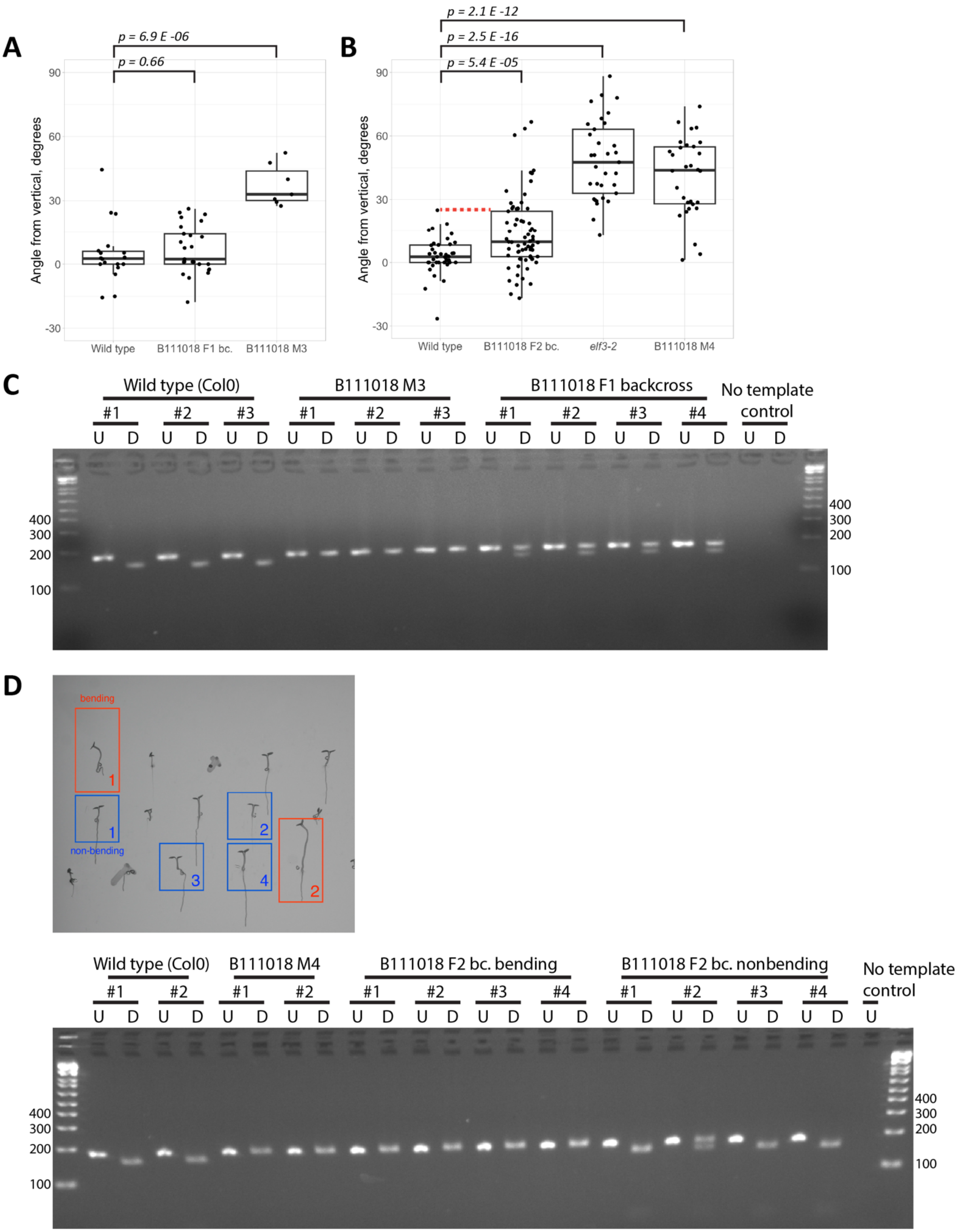
A
p = 6.9 E -06
p = 0.66
Angle from vertical, degrees
90
60
30
0
-30
Wild type
B111018 F1 bc.
B111018 M3
B
p = 2.1 E -12
p = 2.5 E -16
p = 5.4 E -05
Angle from vertical, degrees
90
60
30
0
-30
Wild type
B111018 F2 bc.
elf3-2
B111018 M4
C
Wild type (Col0)
B111018 M3
B111018 F1 backcross
No template control
#1 #2 #3 #1 #2 #3 #1 #2 #3 #4
U D U D U D U D U D U D U D U D U D U D U D
400
300
200
100
D
bending
1
2
1
2
3
4
non-bending
Wild type (Col0)
B111018 M4
B111018 F2 bc. bending
B111018 F2 bc. nonbending
No template control
#1 #2 #1 #2 #1 #2 #3 #4 #1 #2 #3 #4
U D U D U D U D U D U D U D U D U D U D U D U D U
400
300
200
100

Figure S3 (see previous page). Bending in B111018 backcrosses, and genotyping B111018. (A) Quantification of phototropic bending in seedlings of B111018 backcrossed to wild type (F1 generation). Points represent the bending angles of individual seedlings; boxplots show the median bending angle and quartiles. Seedlings were grown in our screen conditions for 5 days, and the bending assay began at dawn on the 6th day. B111018 seedlings (M3 generation) are included as a reference. **(B)** Phototropic bending in seedlings of B111018 backcrossed to WT (F2 generation). *elf3-2* and B111018 seedlings (M4 generation) are included for reference. Note: p-values in (A) and (B) are derived from Welch's t-test. **(C)** Genotyping of B111018 (M3 and F1 backcross). PCR was performed with primers GC146 and GC147, using genomic DNA as a template. The PCR product was either undigested ("U") or digested with XmnI ("D"). The samples were run on a 3% agarose gel at 80V for ~30 min. The PCR product from WT plants digests to 155 + 26 bp fragments, while the 181 bp PCR product from the B111018 mutant is not digested. **(D)** Genotyping of B111018 backcross F2 seedlings. Four F2 seedlings with the increased bending phenotype and four F2 seedlings without this phenotype were selected and genotyped. Top: Sample image of a plate after the bending assay, with some of the seedlings selected for genotyping. Bending (red) and non-bending (blue) seedlings are highlighted. Bottom: Genotyping result, including the seedlings shown in the image of the plate.

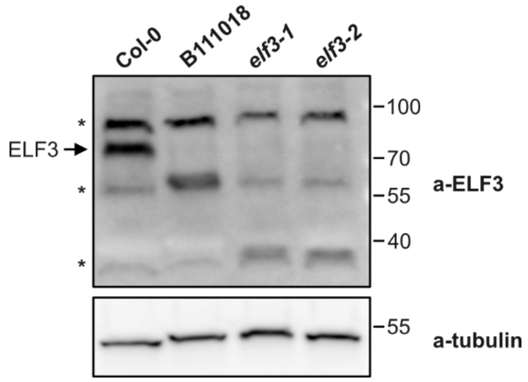

Figure S4. ELF3 protein accumulation in the B111018 allele. Seedlings were grown in 12h light : 12h dark conditions for 7 days. Samples were collected at ZT12. Membrane was probed using native anti-ELF3 antibody (Agrisera AS184168) and anti-tubulin (loading control). ELF3 is indicated with an arrow and asterisks indicate unspecific bands. This experiment was done three times with similar results.

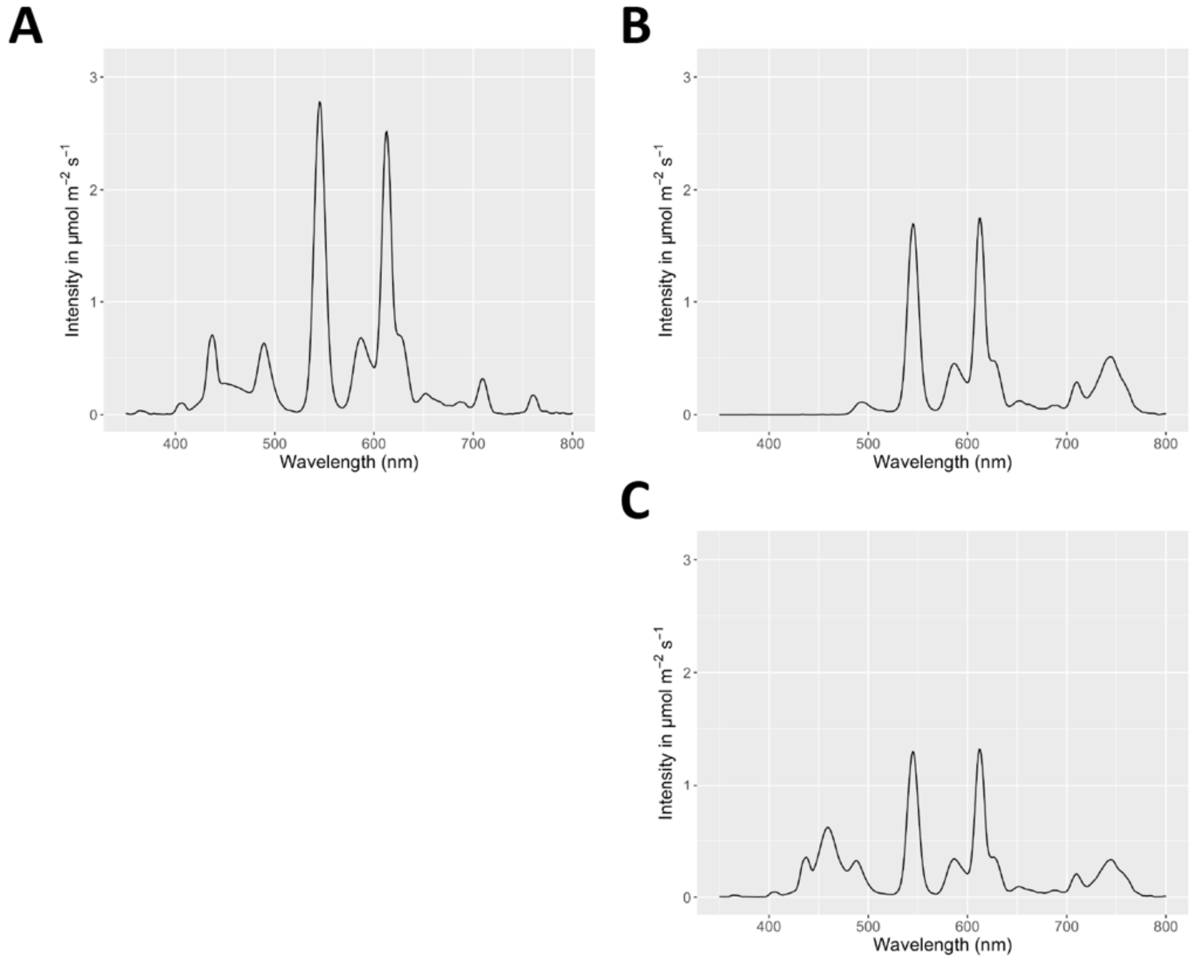

Figure S5. Example spectra of light conditions used in our experiments. (A) Light spectrum used for the standard bending assay. **(B)** Light spectra used for growing seedlings (top) and for inducing phototropic bending (bottom) during the circadian phototropism experiment shown in Figure 4.

A

Line ID number	Blots in which present	Western blot result (M4 seedlings)
I04-0123	#1<br>#4	PHYA expressed in light and dark
B12-1320	#1<br>#3	CRY1 is not expressed
C09-1602	#1<br>#4<br>#5	PHYA expressed in light and dark
C12-0501	#3	CRY1 is not expressed
C06-1423	#1<br>#2<br>#3	Expresses CRY1
C04-1719	#1	Expresses PHYB
B11-1018	#1<br>#4	Expresses CRY1, PHYA, and PHYB

B

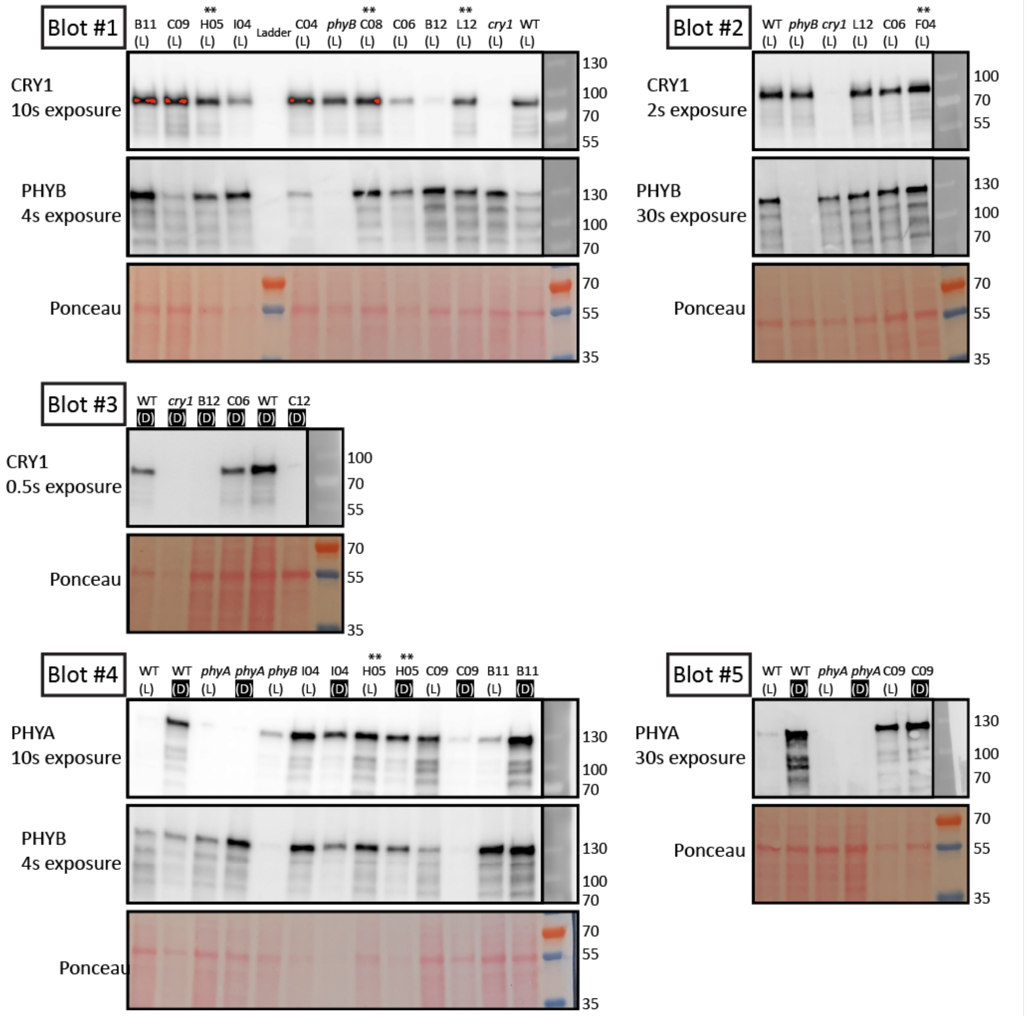

Figure S6. Western blots for PHYA, PHYB, and CRY1 in our candidate lines. (A) Table summarizing the candidate lines, the blots in which they are represented, and our conclusions from the western blots shown here. **(B)** The western blots. Seedlings were grown in the dark for 5 days. They were then either kept in the dark (labelled "D") or were exposed to 6h WL (labelled "L") prior to protein extraction. A color photograph of the membranes stained with Ponceau is shown to give an indication of loading. In cases where a single membrane was probed using multiple antibodies (e.g. blots 1, 2, and 4), it was stripped between probing with the first and second antibody. For more information, see Materials and Methods. Lanes marked with a "**" indicate mutant lines which were not included in our final candidate list. Orange areas in Blot #1 are indicators of overexposure marked by the ImageQuant LAS4000 imaging machine.

Table S1: Primers used in this study.

Part 1: Primers used for genotyping:

Genotyping primers for:	Primers:	Expected amplicon size:
B111018 (*Arabidopsis elf3* allele identified in our screen)	GC146: 5'-ATGAACCAGTTTGGACATCCTGAAAATCTT-3'<br>GC147: 5'-TGCTACCAGAGATTCCCTGTG-3'	Digest the PCR product with XmnI.<br><br>WT gives bands at 155 + 26 bp.<br><br>Mutant gives a band at 181 bp.
elf3-1	OM115: 5'-TGTTGGTCAGTCTTCTCCGA-3'<br>OM116: 5'-TCCCTACTGTCATTCAAGGG -3'	Digest the PCR product with HincII.<br><br>WT gives bands at 500 + 300 bp.<br><br>Mutant gives a band at 800 bp.
elf3-2	GC41: 5'-TGGTTATTTATTCTCCGCTCTTTC-3'<br>GC42: 5'-TTGTTCCATTAGCTGTTCAACCTA-3'<br>GC43: 5'-TGAGTATTTGTTTCTTCTCGAGC-3'<br>GC44: 5'-CATATGGAGGGAAGTAGCCATTAC-3'<br><br>Note: These primers were previously described by Huang et al., 2016.	GC41 + GC42 gives a band of ~400 bp in *elf3-2,* no band in WT.<br><br>GC43 + GC44 gives 579 bp band in the WT, no band in mutant.
elf4-101	GC45: 5'-GCTTCCTATTATATCTTCCCAAATTACCAATACA-3'<br>GC46: 5'-TTCTTGAATCAAAGCAACGTTCTTC-3'<br>GC47: 5'-TTGTAGCTCTTGTCTTGCATAACAT-3'<br><br>GC45 is the standard LB2 primer used for genotyping SAIL lines (See: https://www.arabidopsis.org/abrc/pCSA110.pdf).	GC46 + GC47 gives 804 bp band in WT, no band in mutant.<br><br>GC45 + GC47 gives 563 bp band in the mutant, no band in WT.
lux-4	GC112: 5'-AACACTTAAACGACCGCCATTAGTG-3'<br>GC113: 5'-GTTCTCACGAGTTAATCCTTCAACGTTC-3'	Amplify with GC112 + GC113; digest the PCR product with BstXI.<br><br>WT gives bands at 118 + 24 bp.<br><br>Mutant gives a band at 142 bp.
Luciferase reporters CAB2:LUC and pTOC1:LUC	OM148: 5'-CTCACTGAGACTACATCAGC-3'<br>OM149: 5'-TCCAGATCCACAACCTTCGC-3'	A band of 107 bp occurs if a luciferase reporter is present.
pif4-101	SL42: 5'-CTCGATTTCCGGTTATGG-3'<br>SL43: 5'-CAGACGGTTGATCATCTG-3'<br>oVCG-61: 5'-TAGCATCTGAATTTCATAACCAATCTCGATACAC-3'<br><br>oVCG-61 is the standard LB3 primer used for genotyping SAIL lines (See: https://www.arabidopsis.org/abrc/pCSA110.pdf).	SL43 + oVCG061 gives 870 bp band in the mutant, no band in WT.<br><br>SL42 + SL43 gives 850 bp band in the WT, no band in mutant.
pif5-3	SL46: 5'-TCGCTCACTCGCTTACTTAC-3'<br>SL47: 5'-TCTCTACGAGCTTGGCTTTG-3'<br>oVCG-56: 5'-ATTTTGCCGATTTCGGAAC-3'<br><br>oVCG-56 is the standard LB1.3 primer used for genotyping SALK lines (See: http://signal.salk.edu/tdnaprimers.2.html)	SL46 + oVCG056 gives 860 bp band in the mutant, no band in WT.<br><br>SL46 + SL47 gives 860 bp band in the WT, no band in mutant.

elf3 (Brachypodium distachyon)	GC154: 5'-GGTGGTTTCAGCTTCTGCAGTTGAC-3'<br>GC155: 5'-GCTGTCCCTAGATGCGTGGAATC-3'	Amplify with GC154 + GC155; Digest the PCR product with DdeI.<br><br>WT gives a band at 151 bp.<br><br>Mutant gives bands at 126 bp and 25 bp.<br><br>This *Brachypodium elf3* allele and an alternative dCAPS genotyping strategy were first described by Bouché et al., 2022.

Part 2: Primers used for sequencing:

Fragment	Primers:	Expected amplicon size:
ELF3 #1	GMN029: 5'-AGAGTCCACGTCGTCACGC-3'<br>GMN030: 5'-CACAAGGCTGCATTGAAGAGG-3'	1103 bp
ELF3 #2	GMN031: 5'-AGGATTGAATTCTCCTGTATATATG-3'<br>GMN032: 5'-CTAGTGAACTTACTTGGCAAT-3'	1012 bp
ELF3 #3	GMN033: 5'-AGACATTGATAATGATCGTGAA-3'<br>GMN034: 5'-ATGAGATGTCAGATATTTAACAAG-3'	1118 bp
ELF3 #4	GMN035: 5'-TGAGTCTTATGACGATGCAG-3'<br>GMN036: 5'-TTACCAGGAGGTGGGAAT-3'	1122 bp
ELF3 #5	GMN037: 5'-GCTCCAAATGGATATTGCT-3'<br>GMN038: 5'-GATTTCAAGGCTTTAAGACAA-3'	881 bp
CRY1 #1	CF588: 5'-GACCAAAGGGTTTCGATTTC-3'<br>CF430: 5'-GCGGTTCTTAGAGTATTCAAGC-3'	1067 bp
CRY1 #2	CF306: 5'-CCTTATGACCCTGAGTCTC-3'<br>CF401: 5'-CCTTGGGAACTCTATAGGAGC-3'	1154 bp
CRY1 #3	GC29: 5'-GAGTCCGTTCTTCAAGCTGC-3'<br>GC30: 5'-GCCAACAAGTATGAGACCTGG-3'	1283 bp
PHYB #1	GC35: 5'-TGGGCCCAATCAATCTCTCC-3'<br>GC36: 5'-ACCAGTCAAGTCCCTCACAC-3'	1231 bp
PHYB #2	GC33: 5'-TTCTACTCGAGCGTGCTTTC-3'<br>GC34: 5'-ATTTCCGCAGTTTCCCATGG-3'	1294 bp
PHYB #3	GC31: 5'-GATAGTTTAGGCGATGCGGG-3'<br>GC32: 5'-CCCAATCACTTCACTGCGAG-3'	1024 bp
PHYB #4	PB16: 5'-GAATGCTTGTTCCAGCAAGG-3'<br>PB32: 5'-TGAATTCTGTGCGGATGGCG-3'	1109 bp
PHYB #5	PB23: 5'-ACGCGATTGTAAGTCAAGCG-3'<br>AB48: 5'-TTGGCCTTTACCTCTTGATTGCGT-3'	1259 bp
