# Supplementary material for: Early Flowering 3 (ELF3) Inhibits Hypocotyl Phototropism in Light‐Grown Arabidopsis Seedlings
Source: Plant Direct. 2025 Sep 23;9(9):e70107. doi: 10.1002/pld3.70107 (PMC12455371; doi:10.1002/pld3.70107)
Supplement: Supplementary file 1 — Data S1: Peer review. [file PLD3-9-e70107-s001.pdf]

Plant Direct
Open Access

Early Flowering 3 (ELF3) inhibits hypocotyl phototropism in light-grown Arabidopsis seedlings

Prof. Christian Fankhauser, Dr. Geoffrey M.C. Cobb , Dr. Johanna Krahmer , Dr. Ganesh M. Nawkar , Dr. Alessandra Boccaccini

Decision Letter Round 1:

June 20, 2025

Prof. Christian Fankhauser

Universite de Lausanne

Center for Integrative Genomics

CH-1015 Lausanne .

Switzerland

RE: Early Flowering 3 (ELF3) inhibits hypocotyl phototropism in light-grown Arabidopsis seedlings

Dear Dr. Fankhauser:

Thank you for submitting to Plant Direct. All required reviews have been returned and we have now finished our evaluation of your manuscript. In light of the reviewers' and editor's comments, further revisions are needed before the paper can be accepted for publication in Plant Direct.

Please view reviewers' comments below and use their suggestions as a guide while you work on your revision. In particular, a more detailed description of the experimental set up is needed. The reviewers also have some questions and comments that can help provide a more nuanced discussion of the results.

When uploading the revised version of this article, please be sure to include the following:

-A word document that contains your response to the reviewers. You should respond to each reviewer comment and note the changes made to the manuscript. If you do not agree with a reviewer's comment and choose not to make a suggested revision, please explain why. Please try to provide as complete an answer as possible to each reviewer's criticisms.

-A tracked changes document with each change highlighted

- A clean version of the latest version of the manuscript

To upload your revision, please click the link below.

https://plantdirect.msubmit.net/cgi-bin/main.plex?el=A5Lr6oi3A4pb5I4A9ftdUcpQit6MDlsM6zQEV2WgZ

In order to provide as timely a service as possible, we ask that your revision is resubmitted within three months after receipt of this request. If an extension is needed, please send a request, along with a brief explanation, to the editorial office at plantdirect@wiley.com .

Please note that, in addition to publishing reviewer comments, the author's responses to review comments will also be published alongside the final version of the paper. If you would not like the author's responses to be published, please contact the editorial office at plantdirect@wiley.com .

Thank you very much for giving us an opportunity to review your work. I look forward to receiving the next version.

Sincerely,

Eva Farre

Gustavo MacIntosh

Editor, Plant Direct

------------------------------------------------------------------------------ Editor comments:

------------------------------------------------------------------------------ Reviewer comments:

Reviewer #1:

The manuscript is well written, and the findings presented in the manuscript would be of interest to plant biologists studying the regulation of phototropism and its connection with the circadian network.

In the following section, I will detail some comments related to the criteria determined as relevant by the magazine.

Are the experiments and analysis performed to an acceptable technical standard?

I do consider the technical standard acceptable, but I have some doubts regarding some experimental designs.

I believe that the authors should provide more detailed information regarding the experimental replicates. All physiological figures represent the individual values from the same experiment? If so, the authors should report the number of times each experiment was conducted independently, and if similar results were obtained.

Are the experiments and analysis described in adequate detail in standard English?

The manuscript has been well-written.

One relevant mistake was the use of ZT, as shown in Figures 1 and 3. Under free-running conditions, the corresponding unit is the Circadian Time (CT) and not Zeitgeber Time (ZT).

Also, in figure 3A, it is not clear what they mean with "transfer to constant light, do bending assay". They should rephrase this.

Do the results support the conclusion?

I believe that some of the claims made in the manuscript need to be more firmly established to convincingly support the authors´ conclusions.

First, even though I understand that the results presented regarding the elf3-2 mutants are robust and interesting independent of the initial screening, I believe that the authors could have further proven the identity of the B111018 mutant.

Authors could have measured expression levels of the ELF3 mRNA or the amount of ELF3 protein in this line. Especially given the fact that the point mutation is so close to the C terminus.

Given that the B111018 bending phenotype seems to be much stronger than that in the elf3 mutant, there could be another (most likely linked) mutation in this line that is partially responsible for the phenotype. A complementation assay crossing B111018 and elf3-2 can easily validate this hypothesis.

In addition, I expected a side-by-side bending experiment similar to that performed for leaf movement.

Regarding leaf movement experiments, as a suggestion for future experiments, I would also recommend assaying younger plants for a longer period. In 14-day-old plants, the emergence of the second pair of true leaves tends to attenuate first pair movement. Furthermore, an irradiance over 30-35 umol also tends to dim leaf movement. These comments should be considered, especially when dealing with potentially arrhythmic lines.

Regarding the lack of phenotype in elf4-101 mutants, I wondered if the authors have measured expression levels in this line. Especially considering this mutant line carries a T-DNA insertion in the 5' regulatory region.

From Figure 3, I have several questions.

First, despite the fact that there was a significant reduction in the bending response on the first subjective night of the experiment, I have some concerns regarding the loss of circadian response after just one day under free-running conditions. As most circadian-regulated processes, such as hypocotyl elongation, tend to endure more than two days in continuous light, I wonder if the lack of rhythms through the second day is related to a developmental constraint, for example, the platelet being too old? have you considered transferring the plants to continuous light earlier to assess whether younger plants show a longer sustained circadian phenotype.

On the other hand, I wonder if the authors have an explanation regarding the fact that at CT=0, the elf3 mutant has a stronger bending phenotype under white light (Fig 2A), but a similar response under blue light conditions (Fig 3B).

In addition, could this difference be related to different pulse lengths? (6hs in white light vs 4hs in blue light)

Does the manuscript and contents meet all ethical and research integrity standards?

The manuscript and its contents meet all ethical and research integrity standards.

Reviewer #2:

This is an interesting paper finding a link between the evening complex and PIFs in the regulation of phototropism. The science of the paper is sound and I appreciate the efforts the authors have made in providing graphics to explain their experimental approach. These graphics help massively to understand their paper, which is a non-typical approach to a phototropism screen.

Two recommendations before publication:

Requested change:

The authors propose that EFLs could function redundantly with ELF4. Although an interesting hypothesis, I think such a hypothesis needs correcting as it is not completely accurate. Only EFL1 is capable of restoring the ELF4 circadian phenotype (10.2976/1.3218766). The other EFLs that function outside the ELF4/EFL1 clade cannot rescue the ELF4 circadian phenotype. If the authors are suggesting that their results are part of a clock-regulated process, I would request that the authors amend this part of the discussion or at least clarify it.

Recommendation:

One noticeably lacking element from the paper is a lack of genetic or biochemical characterisation of the new elf3 mutant line. Is it a complete null, i.e. no gene or protein expression? The reason for asking is that this ELF3 mutation could, if expressed, still interact with phyB (ELF3 - phyB interaction is dependent on the N-terminus of ELF3, 10.3389/fpls.2019.00667) and add complexity to the phenotype that they have observed. Have they also tested whether crossing this elf3 mutation into a characterised Col-0 elf3 null mutant results in no change to their observed phenotype? I'm not actively requesting new experiments, but if this data is available, I think this should be included to make a more complete story.

Decision Letter Round 2:

August 22, 2025

Prof. Christian Fankhauser

Universite de Lausanne

Center for Integrative Genomics

CH-1015 Lausanne .

Switzerland

Plant Direct
Open Access

MSID: 2025-01795R1

MS TITLE: Early Flowering 3 (ELF3) inhibits hypocotyl phototropism in light-grown Arabidopsis seedlings

Dear Dr. Christian Fankhauser:

I am pleased to inform you that your manuscript "Early Flowering 3 (ELF3) inhibits hypocotyl phototropism in light-grown Arabidopsis seedlings" has been accepted for publication in Plant Direct.

Your article will appear online in the next available issue of Plant Direct. To ensure your article gets published as quickly as possible, please pay attention to the steps detailed below. We have found that most of the delays happen at this stage, especially at the payment stage, so please respond as quickly as possible when prompted.

License Agreement: Once your article has been accepted it will move to Production and undergo admin and file checking - you may receive an email with any queries we have at this stage. When all required items are received by the publisher and queries resolved, the corresponding author will receive an email from Wiley's Author Services system which will ask them to log on at https://authorservices.wiley.com/bauthor and will present them with the appropriate license for completion. Your article cannot be published until both the signed license agreement and payment of the article fee have been received.

Payment of the Open Access Article Publication Fee: All articles published in Plant Direct are fully open access: immediately and freely available to read, download and share. Plant Direct charges a publication fee to cover the publication costs. The corresponding author for this manuscript should have already received a quote from the payments team (cs-openaccess@wiley.com) with the estimated article publication fee; please email cs-openaccess@wiley.com if this has not been received. The corresponding author should log on to the Wiley Author Services site, where the publication fee can be paid by credit card or an invoice. Pro Forma can also be requested. Payment of the publication charge must be received before the article will be published online.

Proofs: You will have the opportunity to look over your paper once more when you receive the author proofs for your article. The proofs will be with you in approximately two weeks. Please note that, in addition to publishing reviewer comments, the author's responses to review comments will also be published alongside the final version of the paper. If you would not like the author's responses to be published, please contact the editorial office at plantdirect@wiley.com .

Promotion of your article: You can help your research get the attention it deserves! Check out Wiley's free Promotion Guide for best-practice recommendations for promoting your work at www.wileyauthors.com/eeo/guide. And learn more about Wiley Editing Services which offers professional video, design, and writing services to create shareable video abstracts, infographics, conference posters, lay summaries, and research news stories for your research at www.wileyauthors.com/eeo/promotion.

Thank you again for your contribution to Plant Direct. If you have any questions, feel free to contact the editorial office at plantdirect@wiley.com .

Sincerely,

Eva Farre

Gustavo MacIntosh

Editor, Plant Direct

Response Letter Round 1:

Cobb, Krahmer et al., Plant Direct: 2025-01795, response to the reviewers

comments

I would like to thank the reviewers for carefully assessing our mansucript and making

constructive comments to improve it. Please find enclosed our comment in blue to

your specific suggestions & comments

Reviewer #1:

I believe that the authors should provide more detailed information regarding the

experimental replicates. All physiological figures represent the individual values from

the same experiment? If so, the authors should report the number of times each

experiment was conducted independently, and if similar results were obtained.

Thank you for this good suggestion. This information is now provided in the figure

legends.

One relevant mistake was the use of ZT, as shown in Figures 1 and 3. Under freerunning conditions, the corresponding unit is the Circadian Time (CT) and not

Zeitgeber Time (ZT).

We have now updated figures 1 and 3 to correct this mistake

Also, in figure 3A, it is not clear what they mean with "transfer to constant light, do

bending assay". They should rephrase this.

This was indeed unclear and has now been modified in the figure and clarified in the

figure legend.

First, even though I understand that the results presented regarding the elf3-2

mutants are robust and interesting independent of the initial screening, I believe that

the authors could have further proven the identity of the B111018 mutant.

Authors could have measured expression levels of the ELF3 mRNA or the amount of

ELF3 protein in this line. Especially given the fact that the point mutation is so close

to the C terminus.

This is a good suggestion and we now present a western blot comparing the WT, our

elf3 allele (B111018), elf3-1 and elf3-2 in figure S4. The elf3 B111018 allele

accumulates a protein with a size fitting the expected truncated ELF3. Importantly,

this truncated ELF3 lacks the putative ELF3 NLS (Liu et al., 2001) and the the Cterminus of ELF3 was previously shown to be required for robust nuclear

accumulation of the protein and ELF3 function (Herrero et al., 2012). Our data

therefore confirms earlier findings indicating the functional importance of ELF3's Cterminus. This is now discussed in the revised manuscript.

Given that the B111018 bending phenotype seems to be much stronger than that in

the elf3 mutant, there could be another (most likely linked) mutation in this line that is

partially responsible for the phenotype. A complementation assay crossing B111018

and elf3-2 can easily validate this hypothesis.

2

In addition, I expected a side-by-side bending experiment similar to that performed

for leaf movement.

Thanks for allowing us to clarify this. The phototropic phenotype of the elf3 B111018

allele and elf3-2 is directly compared in figure S3B. elf3 B111018 has a slightly but

not siginificantly weaker phenotype than elf3-2 (figure S3B). Hence both for the leaf

movements (Figure 1C) and for phototropism (Figure S3B) elf3 B111018 was similar

to the presumptive elf3 null alleles. We feel that this establishes the mutation in the

ELF3 gene as causative to the phototropic defect in B111018 and indicates that this

is a strong loss of function allele. See also answer to the previous point.

We also note that the the phenotype of B111018 looks stronger in figure 1A than in

S3B. These phototropic assays are a bit variable which is why we always include a

wild type control (and if needed other controls) in the different experiment and only

compare absolute values within experiment done at the same time. The likely

variable include, how dry is the surface of the plates, exact developmental age of the seedlings which influences hypocotyl length & phototropic potential. Differences in germination time of a few hours have effects that we cannot fully control despite using highly standatised procedures.

Regarding leaf movement experiments, as a suggestion for future experiments, I would also recommend assaying younger plants for a longer period. In 14-day-old plants, the emergence of the second pair of true leaves tends to attenuate first pair movement. Furthermore, an irradiance over 30-35 umol also tends to dim leaf movement. These comments should be considered, especially when dealing with potentially arrhythmic lines.

Thank you for this suggestion. We have previously also noted and published that leaf movements decrease with leaf age. This experiment was a test to determine whether the B111018 mutant has circadian defects comparable to the one of elf3. Given that the result was very clear, we feel that repeating this with younger seedlings is not required.

Regarding the lack of phenotype in elf4-101 mutants, I wondered if the authors have measured expression levels in this line. Especially considering this mutant line carries a T-DNA insertion in the 5' regulatory region.

This allele was described in Khanna et al., 2003 and the authors report that ELF4 transcripts were undetectable. We have indicated this in the manscript. Understanding the precise function of ELF4 in the EC is not a focus of our mansucript, we therefore did not conduct additional experiments regarding ELF4. However, in response to a comment of reviewer 2, we modified the discussion about ELF4.

From Figure 3, I have several questions.

First, despite the fact that there was a significant reduction in the bending response on the first subjective night of the experiment, I have some concerns regarding the loss of circadian response after just one day under free-running conditions. As most circadian-regulated processes, such as hypocotyl elongation, tend to endure more

than two days in continuous light, I wonder if the lack of rhythms through the second

3

day is related to a developmental constraint, for example, the platelet being too old? have you considered transferring the plants to continuous light earlier to assess whether younger plants show a longer sustained circadian phenotype.

Thanks for this comment for which we can offer the following explanations. The amplitude of this rhythm is modest, hence with dampening which happens for circadian rhythms in free running conditions this may explain why we rapidly can't detect it any longer. Moreover, we note that in potato plantlets, Vinterhalter and colleagues made exactly the same observation. We therefore feel that our observation is not unexpected. Playing with developmental age of the seedlings and/or precise growth conditions may allow to detect such rhythms over a longer time but given how involved these experiments are, we have not attempted this further.

On the other hand, I wonder if the authors have an explanation regarding the fact that at CT=0, the elf3 mutant has a stronger bending phenotype under white light (Fig 2A), but a similar response under blue light conditions (Fig 3B).

Our model is that in white light conditions ELF3 contributes to inhibiting PIFs and hence in an elf3 mutant (as in a phyB mutant) phototropism in enhanced. This is consistent with our previous work and with the genetic interaction between ELF3 and PIF mutants shown in Figure 2. In contrast, in shade conditions, as used in Figure 3B, the inhibition of PIFs is released allowing for a robust phototropic response also in the wild type. Hence in shade lacking ELF3 function does not lead to enhanced phototropism.

In addition, could this difference be related to different pulse lengths? (6hs in white light vs 4hs in blue light)

The regulation by shade of phototropism (see also Goyal et al., 2016 and Boccaccini et al., 2020) provide a good explanation for this condition-depend phenotype of elf3.

Reviewer #2:

The authors propose that EFLs could function redundantly with ELF4. Although an interesting hypothesis, I think such a hypothesis needs correcting as it is not completely accurate. Only EFL1 is capable of restoring the ELF4 circadian phenotype (10.2976/1.3218766). The other EFLs that function outside the ELF4/EFL1 clade cannot rescue the ELF4 circadian phenotype. If the authors are suggesting that their results are part of a clock-regulated process, I would request that the authors amend this part of the discussion or at least clarify it.

We thank the reviewer for pointing out this publication. We have modified the discussion to take this into consideration and cited the suggested paper.

One noticeably lacking element from the paper is a lack of genetic or biochemical characterisation of the new elf3 mutant line. Is it a complete null, i.e. no gene or protein expression? The reason for asking is that this ELF3 mutation could, if expressed, still interact with phyB (ELF3 - phyB interaction is dependent on the Nterminus of ELF3, 10.3389/fpls.2019.00667) and add complexity to the phenotype

4

that they have observed. Have they also tested whether crossing this elf3 mutation into a characterised Col-0 elf3 null mutant results in no change to their observed phenotype? I'm not actively requesting new experiments, but if this data is available, I think this should be included to make a more complete story.

Please also check our answer to reviewer 1. This elf3 mutant accumulates a protein consistent with the size of the truncated ELF3 (early stop). See new Figure S4. We note that this truncated ELF3 lacks the putative NLS described in Liu et al 2001. Moreover, a subsequent publication from the Davis lab showed that the C-terminus of ELF3 is required for robust nuclear import and ELF3 function (Herrera et al., 2012). Given that this allele has the same phenotype as presumptive null alleles of elf3 for leaf movements (Figure 1C) and phototropism (Figure S3B) and considering the work of Herrera et al., 2012, we believe that this mutant is a strong loss of function allele. This has now been clarified in the results and discussion.
